# PolyID: Artificial Intelligence for Discovering Performance-Advantaged and Sustainable Polymers

**DOI:** 10.1021/acs.macromol.3c00994

**Published:** 2023-10-19

**Authors:** A. Nolan Wilson, Peter C. St John, Daniela H. Marin, Caroline B. Hoyt, Erik G. Rognerud, Mark R. Nimlos, Robin M. Cywar, Nicholas A. Rorrer, Kevin M. Shebek, Linda J. Broadbelt, Gregg T. Beckham, Michael F. Crowley

**Affiliations:** † Renewable Resources and Enabling Sciences Center, National Renewable Energy Laboratory, 15013 Denver West Parkway, Golden, Colorado 80401, United States; ‡ Department of Chemical and Biological Engineering and Center for Synthetic Biology, Northwestern University, Evanston, Illinois 60208, United States; § Chemistry of Life Processes Institute, Northwestern University, Evanston, Illinois 60208, United States

## Abstract

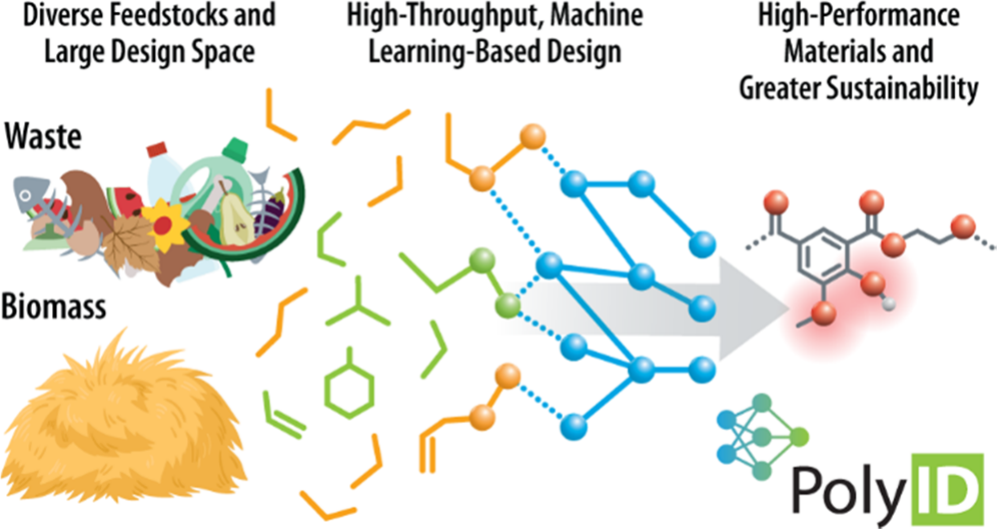

A necessary transformation for a sustainable economy is the transition from fossil-derived plastics to polymers derived from biomass and waste resources. While renewable feedstocks can enhance material performance through unique chemical moieties, probing the vast material design space by experiment alone is not practically feasible. Here, we develop a machine-learning-based tool, PolyID, to reduce the design space of renewable feedstocks to enable efficient discovery of performance-advantaged, biobased polymers. PolyID is a multioutput, graph neural network specifically designed to increase accuracy and to enable quantitative structure–property relationship (QSPR) analysis for polymers. It includes a novel domain-of-validity method that was developed and applied to demonstrate how gaps in training data can be filled to improve accuracy. The model was benchmarked with both a 20% held-out subset of the original training data and 22 experimentally synthesized polymers. A mean absolute error for the glass transition temperatures of 19.8 and 26.4 °C was achieved for the test and experimental data sets, respectively. Predictions were made on polymers composed of monomers from four databases that contain biologically accessible small molecules: MetaCyc, MINEs, KEGG, and BiGG. From 1.4 × 10^6^ accessible biobased polymers, we identified five poly(ethylene terephthalate) (PET) analogues with predicted improvements to thermal and transport performance. Experimental validation for one of the PET analogues demonstrated a glass transition temperature between 85 and 112 °C, which is higher than PET and within the predicted range of the PolyID tool. In addition to accurate predictions, we show how the model’s predictions are explainable through analysis of individual bond importance for a biobased nylon. Overall, PolyID can aid the biobased polymer practitioner to navigate the vast number of renewable polymers to discover sustainable materials with enhanced performance.

## Introduction

Replacing fossil-based with biobased plastics can play a key role in developing a circular materials economy and in reducing greenhouse gas (GHG) emissions from polymer manufacturing, which are expected to grow from 5 to 15% of the global carbon budget from 2015 to 2050.^1,2^ Market penetration of biobased polymers is less than 1% of the plastics market with polylactic acid possessing the largest production volume at 282 kilotonnes annually as of 2021.^3,4^ Increasing the adoption rate of polymers containing biobased monomers will help meet climate and sustainability goals and can be driven by improvements to properties critical to performance and production at competitive pricing.^5−7^

Balancing performance across multiple material properties remains a challenge in polymer discovery and redesign.^8^ By leveraging the inherent chemical functionality afforded by biobased feedstocks, it is possible to improve polymer properties to optimize material performance and ultimately drive market adoption.^5,9,10^ However, the design space for material discovery is vast with monomers accessible from biological and chemical transformations of biobased feedstocks exceeding >1 × 10^5^, which can be combined in a combinatorial number of polymers. Thus, there is a clear need for rapid and accurate property prediction tools to facilitate the development of biobased polymers.

High-throughput machine learning tools can provide an accelerated, data-driven approach to material discovery, including for sustainable polymers.^11,12^ Polymer property prediction based on molecular structure has evolved from group contribution theory to advanced molecular descriptors.^13−16^ However, these descriptors use static featurization kernels or rules to abstract the chemical environment. Modern data science techniques can be applied to better “featurize” biobased polymers that exhibit unique chemical functionality relative to traditional polymers. To this end, recent advances have extended the featurization task to enable “end-to-end” learning on molecules.^17,18^ End-to-end learning allows for both feature extraction and prediction to be handled simultaneously, and these methods achieve state-of-the-art prediction accuracies for both small-molecule and polymer properties.^19−21^

In this study, we developed an end-to-end learning, multioutput, message-passing neural network, PolyID, that was specifically designed for polymer prediction. We ensured confidence in prediction accuracy through experimental validation. An intuitive and interpretable domain-of-validity method is developed to ensure relevant training data are used for the desired prediction task. As an illustrative example of biobased polymer discovery, we used property predictions and the developed domain-of-validity method to screen for performance-advantaged replacements of poly(ethylene terephthalate) (PET) from 1.4 × 10^6^ biobased polymers, yielding five potential candidates. One of the five PET replacements were synthesized experimentally and demonstrated properties close to model predictions. Finally, we show how structure–property relationships can be explored by using the developed message-passing network. Using end-to-end machine learning methods and experimental validation, this work demonstrates that the discovery of performance-advantaged biobased polymers can be catalyzed by leveraging data science.

## Results

### Development of a Polymer Property Prediction Tool

**Figure 1:**
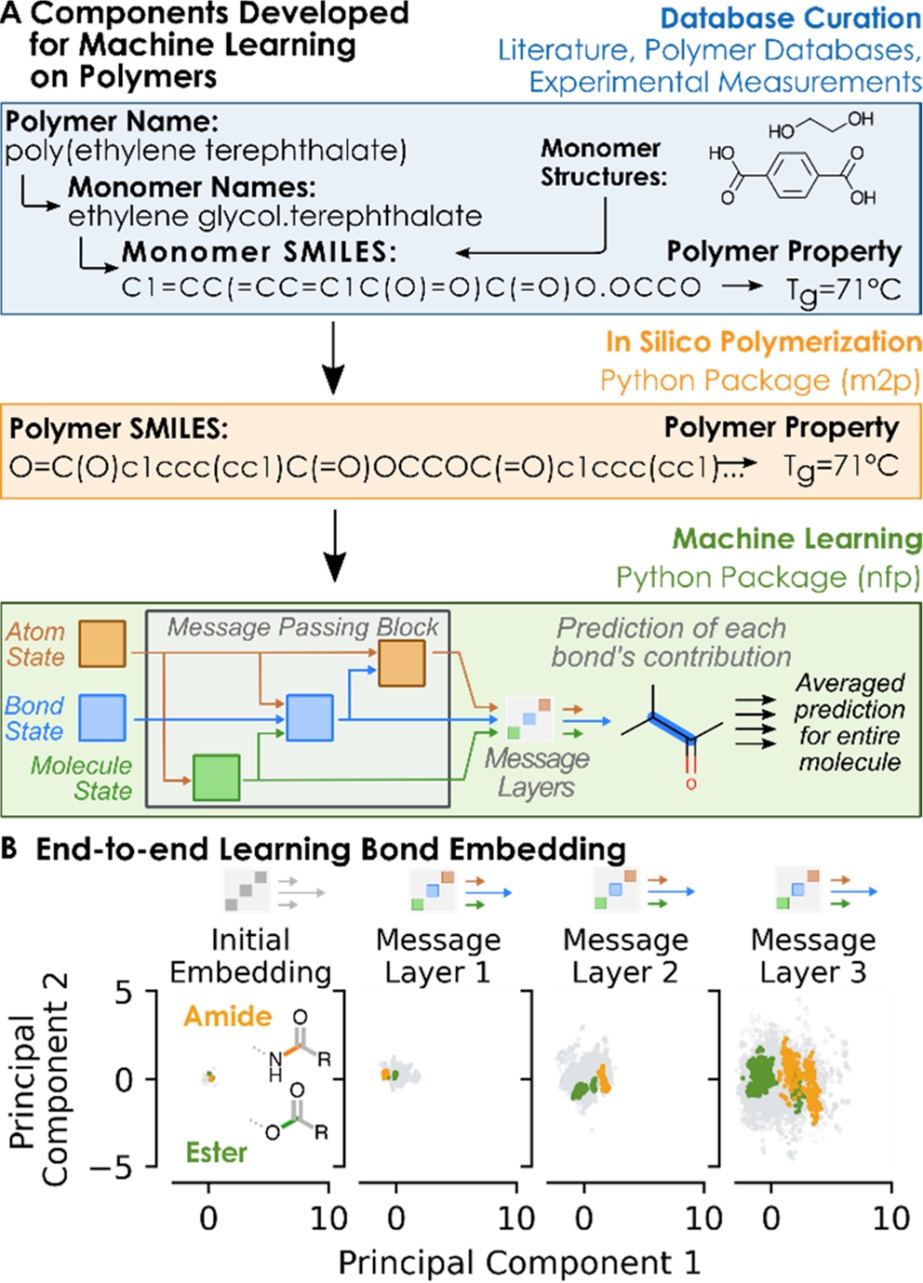
(A) Components required for the machine learning tool included a training set where the monomer structure was mapped to the polymer property, an *in silico* polymerization scheme, and a message-passing neural network for prediction. (B) Principal components of the bond feature vector as the polymer structures are passed through each layer show greater bond differentiation as the message passing proceeds. Amide, ester, and all other bonds are shown in green, orange, and gray, respectively.

To build and apply the machine learning tool, three components were needed: (1) a labeled database with polymer properties and a prediction database of bioaccessible monomers, represented using the Simplified Molecular-Input Line-Entry System (SMILES), (2) *in silico* polymerization schemes for automated generation of high-fidelity polymer structures from the monomer SMILES, and (3) a message passing neural network architecture tuned for polymer property prediction, all of which are shown in Figure 1A. Details for the curation and splitting of the labeled database, containing 1791 unique polymers, and for the curation of the prediction database are provided in the Methods section. The curated data set contains 8 polymer properties: glass transition temperature (*T*_g_), melt temperature (*T*_M_), density (ρ), modulus (*E*), and the permeability of O_2_, N_2_, CO_2_, and H_2_O (*P*_M-O_2__, *P*_M-N_2__, *P*_M-CO_2__, *P*_M-H_2_O_). During message passing, atom, bond, and molecular “states” are initialized as one-hot encodings, and these vectors differentiate themselves during message passing based on local environments, which is ideal for embedding chemical structures as they are affected by this environment. From Figure 1B, increased differentiation of ester and amide bonds in the latent space is visible as message passing proceeds through the network, and further analysis of the ester bonds revealed clustering based on polymer type, as shown in Figure S1.

To develop PolyID specifically for polymers, we made design decisions around the structural representation of the polymer and the network architecture to improve performance and interpretability. We show how larger representations of polymer structures in conjunction with deeper message-passing neural networks can be used to improve network accuracy. Often neural networks are seen as “black-box” tools; therefore, we constructed the network architecture to enable structure–property relationship interpretation by pooling each bond into a single value at the end of the network and making the sum over all bond values result in the predicted property. Finally, a domain validity method was developed as part of the tool to ensure the accuracy of the predicted values. To validate the tool, we experimentally synthesized and characterized the thermal properties of 10 polyesters and 12 polyamides in addition to withholding a random 20% of our polymer database as a test set (vide infra).

### Considerations for Using AI in Polymer Discovery

**Figure 2:**
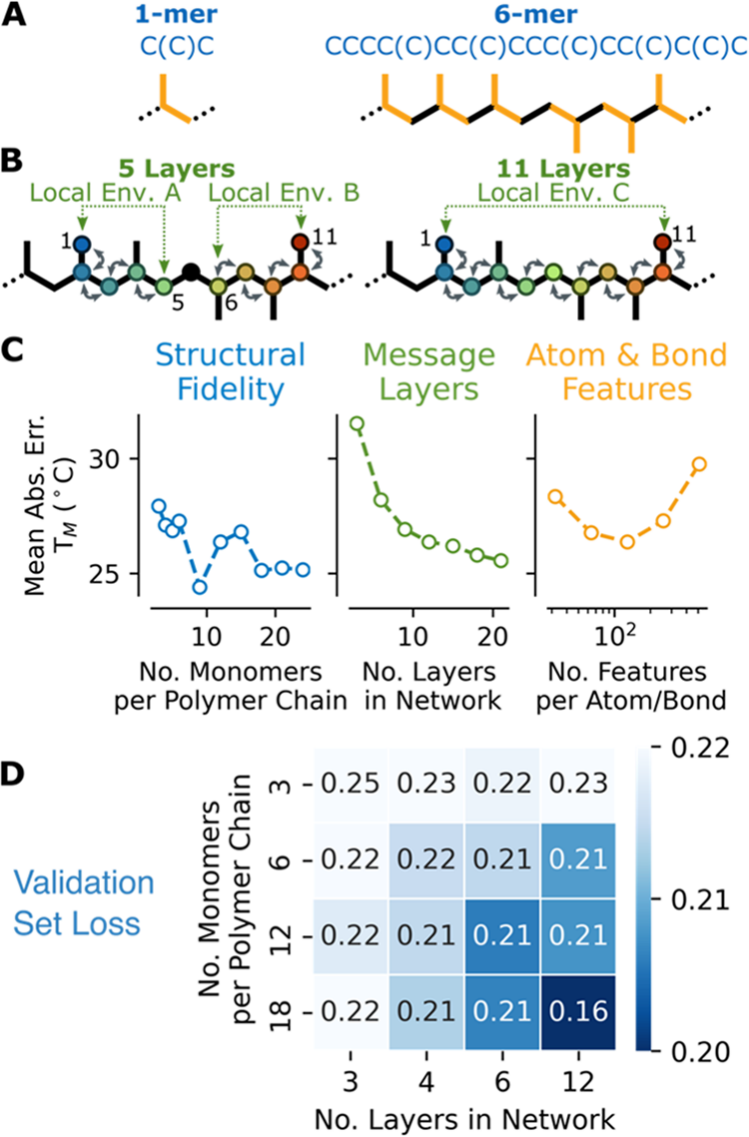
(A) Ability to incorporate regiovariability in the SMILES representation of polypropylene is shown by increasing the number of monomer units from 1 to 6. (B) Increasing the structural fidelity and message layers reduces error. When the number of message-passing layers is 5, atoms 1 and 5 (local environment A) as well as atoms 6 and 11 (local environment B) can share information, but atoms 1 and 11 do not share information. When the number of message layers is increased to 11, the local environment is expanded, and atoms 1 and 11 can share information (local environment C). (C) Increasing monomers in the polymer chain and the number of message-passing layers reduces error. Optimizing atom and bond feature vector length reduces error. Numerical data for this figure are provided in Table S3. (D) Validation set loss (low is better) as a function of network depth and polymer size shows that as polymer size is increased, network depth must also be increased to improve performance.

End-to-end learning approaches for polymers should consider both the structural representation of the polymer and the network architecture to improve accuracy, which is demonstrated in Figures 2A–C and S2. Details for the generation of these figures are provided below Figure S2. Rather than representing polymer structures as simple repeat units, which does not capture random comonomer order or regio- orientations in the polymer, we react monomer SMILES into detailed polymer structures to better represent the structural heterogeneity present in the polymer. For training and prediction tasks, the polymer chain length and the number of polymer chains, “replicates”, can be increased or decreased to capture the multitude of monomer configurations found in a polymer structure and balance computational expense against prediction accuracy. The *in silico* polymerization scheme is therefore capable of incorporating structural differences that arise from random comonomer insertion, random regio-orientation, and varying comonomer composition. Details of the polymer structure building algorithm are provided in the Methods section. Figure 2A compares a 1-mer and 6-mer of polypropylene where the former has no structural heterogeneity and the latter has structural heterogeneity that arises from random regio-orientation. Results in Figures 2C and S2 demonstrate that the prediction error can be reduced by moving beyond simple repeat unit representations to representations with greater structural fidelity. Performance can also be improved by increasing the number of message-passing layers, which increases the size of the environment from which an atom or a bond can infer chemical information, as shown in Figure 2B. However, improvements to prediction accuracy as a function of the number of message-passing layers eventually saturates, as shown in Figures 2C and S2. The saturation of performance could be attributed to oversmoothing or oversquashing, which are known issues in graph networks, and further increasing of network depth could eventually degrade performance.^22,23^ Atom and bond feature vector lengths have an optimal range, as shown in Figures 2C and S2, which is likely due to a trade-off between bias and variance for a given training database size, network size, and regularization. Figures 2D and S3 demonstrate the relationship between polymer size and network depth. As the number of monomers in a polymer chain is increased, the depth of the message-passing network must also increase to encode additional structural information across larger and more diverse local environments. Table S1 provides the optimized hyperparameters that were used to make the predictions in this work. While others have shown multitask can outperform single-task performance in polymer property prediction, Table S2 shows no consistent performance improvement when the number of properties used in training are increased.^24,25^ By considering the trends from polymer chain length and message layer depth, we show that end-to-end learning on polymers can be improved through improved structural representation and by increasing network depth.

### Validating Predictions through Domain of Validity, Test Sets, and Experiment

Before using the polymer prediction tool to identify performance-advantaged biobased polymers, we aimed to validate the model and ensure confidence in the predicted values through two approaches. First, we confirmed prediction structures were in a similar chemical domain as the training set used to parametrize the model by developing an interpretable, structure-based domain of validity method. Second, we synthesized new polymers that were not in the training set, measured their properties, and compared the experimental and predicted values.

**Figure 3:**
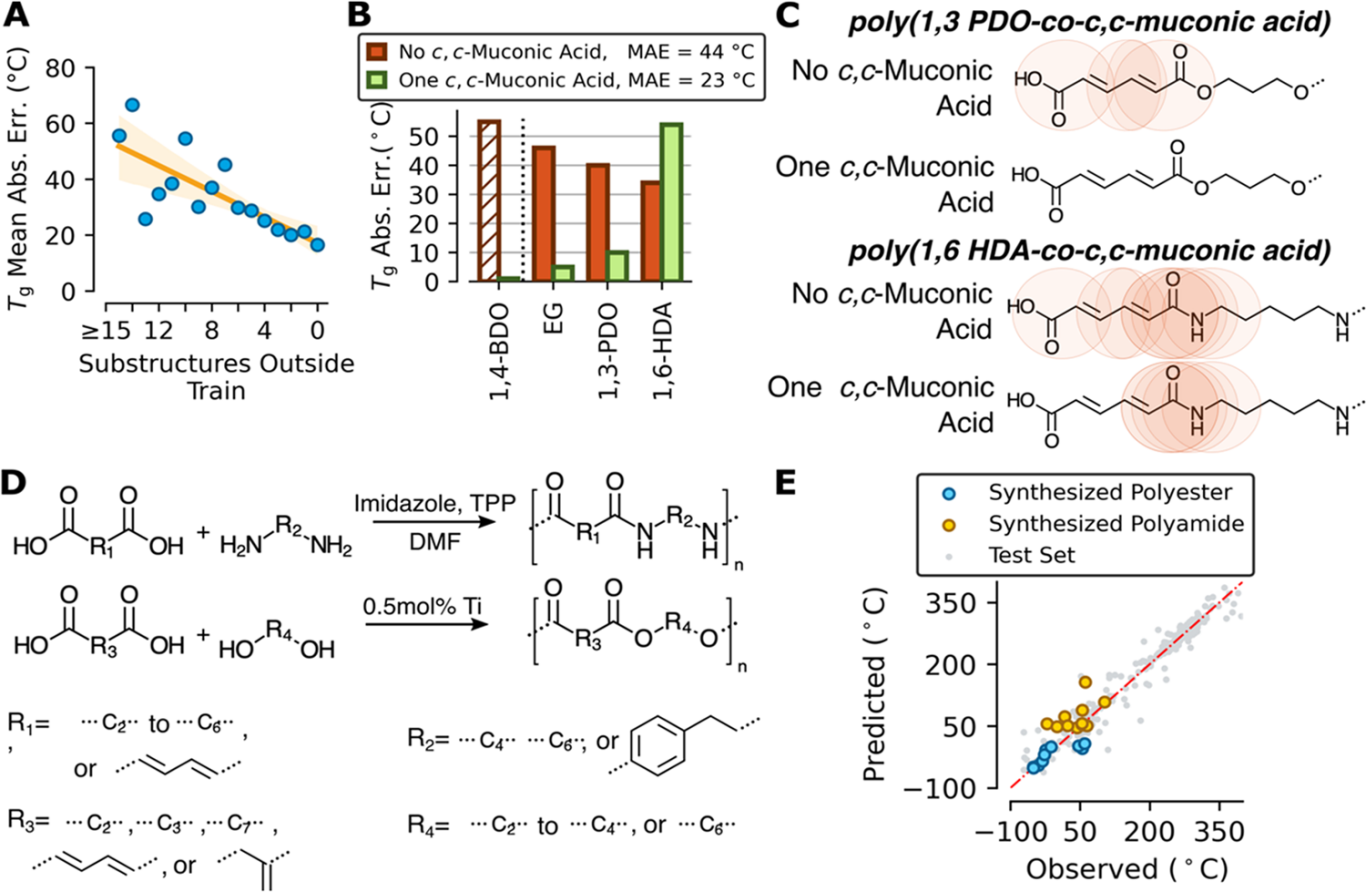
(A) Mean absolute error (MAE) decreases as the level of substructure overlap between training and prediction structures increases. Numerical data are provided in Table S5. (B) Glass transition prediction accuracy for *c,c*-muconic acid-containing polymers when the training set contains no *c,c*-muconic acid-based polymers (red) vs when it contains one *c,c*-muconic acid-based polymer (green). Co-monomers used with *c,c*-muconic acid were ethylene glycol (EG), 1,3-propanediol (1,3-PDO), and 1,6-hexanediamine (1,6-HDA). The dashed bar is the data point that was added to the training set while the other *c,c*-muconic acid-based polymers were in neither training set. Numerical data are provided in Table S6. (C) Circled substructures not contained in the training set when no *c,c*-muconic acid-containing polymers are in the training set vs one *c,c*-muconic acid-containing polymer is in the training set. (D) Experimental synthesis schemes for polyesters and polyamides. (E) Parity plot for the glass transition temperature of predicted and experimentally determined values for synthesized polyesters and polyamides and test set data. Numerical data are provided in Table S7.

The domain of validity method that was developed sums the number of Morgan fingerprints (substructures) in a predicted polymer that are not found in the training set.^26,27^Figure 3A shows that the mean absolute error is reduced for structures with high overlap in substructures with the training data. When a target polymer contains many substructures that are not found in the training data, then the predictions should be disregarded. Based on the observed increase in the variance above seven substructures outside the training set in Figure 3A, a value of seven was selected as the threshold for which polymers were considered outside the domain of validity for this work.

Building on this domain of validity method, we used two separately trained models to show how prediction accuracy is substantially impacted by the structural overlap between the training and prediction sets. One model was trained on a database with no polymers containing *cis,cis-*muconic acid (*c,c*-muconic acid), a biobased monomer,^28^ and the other model was trained on a database with a single instance of a *c,c-*muconic acid-containing polymer in the training set, poly(1,4-butanediol-*co*-*c,*c*-*muconic acid).^29^ By adding a single, task-relevant data point to the training set, the mean absolute error of the glass transition temperature (*T*_g_) for three *c,c-*muconic acid-containing polymers excluded from the training set was reduced from 40 to 23 °C, as shown in Figure 3B. Upon addition of the *c,c-*muconic acid-containing polymer, the chemical substructures outside the training set dropped from 7 to 4 and from 4 to 0 for the polyamide and all polyesters, respectively. The only polymer with a worse mean absolute error, poly(1,6-hexamethylenediamine-*co*-*c,*c*-*muconic acid), contains amide linkages with conjugation to an unsaturated bond, which is a substructure that was still not in the training set, Figure 3C. Targeted augmentation of the database to incorporate an amide linkage next to an unsaturated bond would likely further improve the predictive performance. Substructure analysis enabled by the developed domain of validity method can easily identify task-relevant data for targeted data extraction.^30,31^

To validate the accuracy of the trained network, we used a 20% holdout test set, and additionally, we experimentally synthesized and characterized 22 polymers (10 esters and 12 amides) that were not in the database. Table S4 contains the validation set and the test set mean absolute errors. Figure 3D shows the general synthesis scheme for producing polyesters and polyamides. The parity plot in Figure 3E shows the experimental and predicted glass transition temperatures for the synthesized materials and test set. For the experimentally synthesized polymers, Table S7 and Figure S4 contain the thermal data and selected nuclear magnetic resonance (NMR), respectively. Model predictions for synthesized polyesters and polyamides were slightly less accurate than predictions for the same polymer classes in the held-out test set, with a mean absolute error in *T*_g_ of 26.4 and 17.3 °C for the experimental and test sets, respectively. This was expected, as the average number of the substructures outside the training set was 1.6 for the experimental set and 0.5 for the held-out test set. With the experimentally validated model and domain of validity method in-hand, the machine learning tool was then applied to discover new performance-advantaged biobased polymers.

### Coupling Machine Learning with Metabolic Modeling to Discover Performance-Advantaged Biobased Polymers

**Figure 4:**
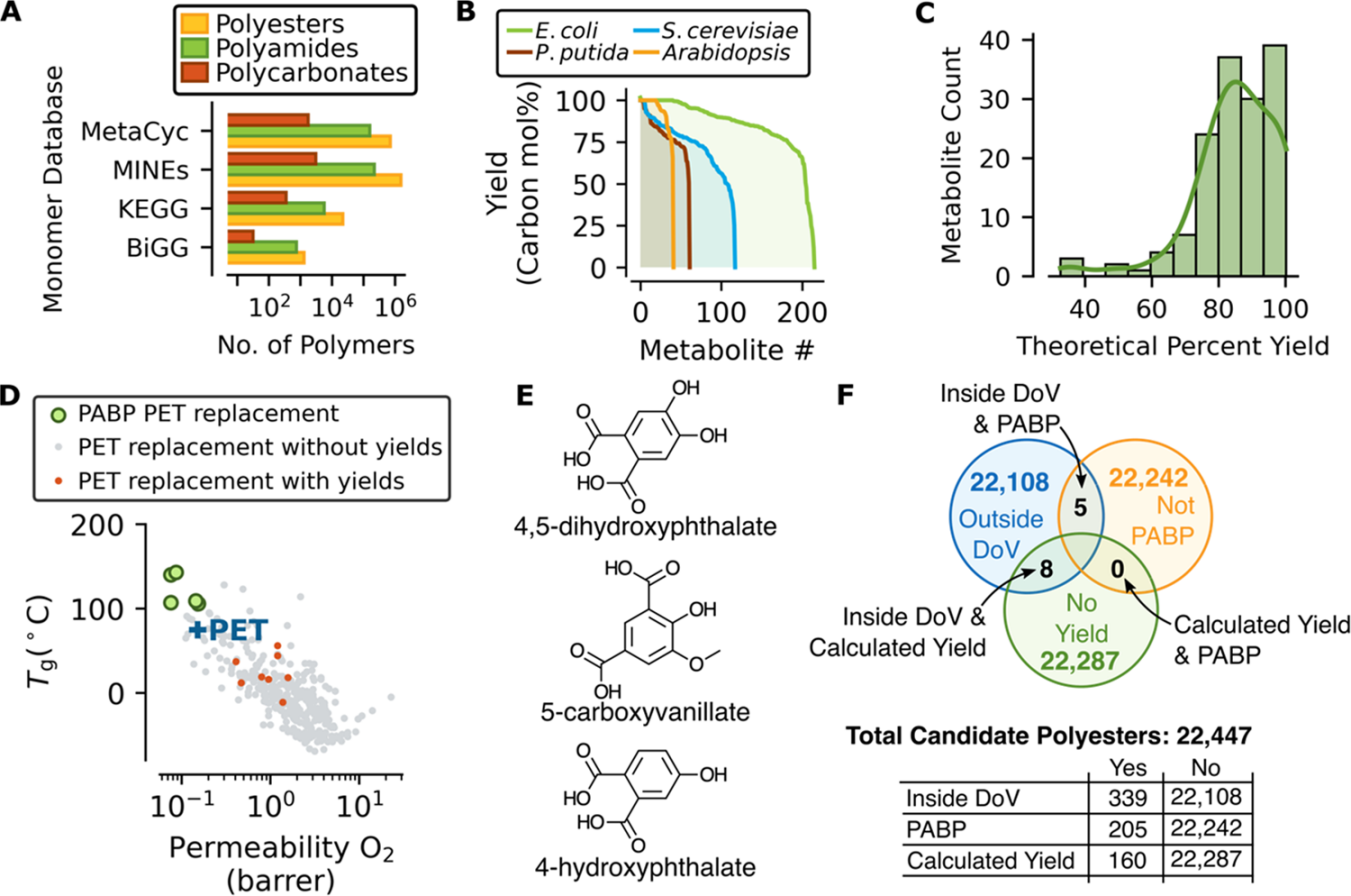
(A) Combinatorial set of polyesters, polyamides, and polycarbonates synthesized from four databases of metabolites. Numerical data are provided in Table S9. (B) Theoretical yields for bioaccessible metabolites based on BiGG metabolic flux models. (C) Histogram of the theoretical percent yield for 55 metabolites that can participate in polyester, polyamide, or polycarbonate synthesis. (D) Screening for performance-advantaged biobased (PABP) PET replacements via scatter plot of oxygen permeability and glass transition temperature. Green dots indicate polyesters predicted to be PABP PET replacements within the domain of validity (DoV). Red circles indicate polyesters with determined theoretical yields within the DoV. Gray dots indicate polyesters within the DoV that were predicted to not be PABP. (E) Diacids were from candidate PET replacements. (F) Venn diagram and inset table showing breakdown of polyesters based on DoV criteria, PABP criteria, and calculatable theoretical yields.

The discovery of biobased polymers can be enabled by coupling computational methods that identify performance-advantaged polymers with methods based on metabolic models that predict efficient routes for their production. Figure 4A shows a lower bound for the number of potential biobased polymers that was determined for the polyesters, polyamides, and polycarbonates that could be synthesized from compounds in four metabolite databases.^32−35^

To identify monomers that can be produced efficiently, constraint-based metabolic reconstruction and analysis was used to determine theoretical yields of analytes that could be synthesized into biobased polymers.^36,37^ Calculated theoretical yields for annotated metabolites are shown in Figure 4B from glucose in *Escherichia coli* (*E. coli*), *Saccharomyces cerevisiae* (*S. cerevisiae*), and *Pseudomonas putida* KT2440 (*P. putida*) and from CO_2_ in *Arabidopsis thaliana*.^38−40^ It was found that 102 of the 328 analytes with determined theoretical yields had the necessary functionality to participate in polyester, polyamide, or polycarbonate condensation chemistries. Of these, 55 were predicted to exhibit theoretical yields above 90%, which provides a subset of metabolites with the potential for carbon-efficient production. Figure 4C shows the distribution in the theoretical percent yield for the 147 metabolites. For comparison, lactic acid, which is produced at commercial scale through fermentation, has a theoretical yield of 100% from glucose, and metabolic engineering efforts have achieved a 93% yield in practice.^41^ Numerical data for theoretical yields are provided in Table S8.

To validate our predictions, a performance-advantaged biobased polymer (PABP) analogue of PET was targeted with a *T*_g_ above the boiling point of water and with equivalent or improved O_2_ barrier properties. PET is the world’s largest condensation polymer with a market of 26 million tons in 2021, and its primary uses include carpets, clothing, single-use beverage bottles, and food packaging.^42,43^ Improvements to thermal or barrier properties of biobased PET replacements could expand its application domain and drive market adoption through superior performance. Using monomers from the KEGG database that met the prescreening criteria described in the Methods section, we screened 22,447 polyester candidates and identified 5 polymers that met the domain of validity and desired performance criteria based on predicted performance, which are plotted in Figure 4D (green circles) and illustrated with associated data in Figure S5.^32^ The improved material properties can be attributed to 3 diacids, 4,5-dihydroxphthalate, 4-hydroxpthalate, and 5-carboxyvanillate, which are shown in Figure 4E. The diacids are structural analogues of phthalic and isophthalic acid with additional oxygen functionalities imparted from biochemical transformations that likely contribute to enhanced predicted performance. Investigation of the biosynthetic pathways found that the phthalate analogues have been implicated in polycyclic aromatic hydrocarbon and phthalate catabolism while 5-carboxyvanillate is found in the lignin biphenyl pathways.^44−46^ A standard and an extended synthesis protocol, which is described in the Methods section, is performed to synthesize poly(ethylene 5-carboxyvanillate) (PEC). The standard protocol had a shorter reaction time that produced lower molecular weight material with a *T*_g_ of 85 °C. For the extended protocol that had a longer reaction time, a higher degree of conversion was achieved that produced insoluble product with a *T*_g_ of 112 °C. The extended protocol produced a polymer with a *T*_g_ value within the predicted range of 106 ± 9 °C, which is higher than the *T*_g_ of PET. The model could not capture the molecular weight dependence of the *T*_g_. This dependence is described by the Flory–Fox equation and could be incorporated into future, physics-informed prediction models. The synthesis of PEC proved to be challenging as the phenoxy and methoxy groups likely contributed to recalcitrant polymerizations, resulting in difficulties with standard polyester synthesis techniques. For further details, the characterization data and analysis are provided in Figure S6A–F.

Results from the yield analysis, property prediction, and domain of validity screening reveal opportunities to improve our ability to combine computational tools for the discovery of performance-advantaged biobased polymers. Figure 4F shows that while 205 of the 22,447 candidate polyesters from the KEGG database could outperform PET, only 5 of them were within the domain of validity. Similarly, only 8 of the 339 polymers had yields that could be calculated using the available metabolic models for both monomers used in the polymerization reaction and were within the domain of validity, which are provided in Table S10 and are shown as red dots in Figure 4D. The table inset of Figure 4F indicates how many of the 22,447 candidates were (yes) or were not (no) inside the DoV, PABP, or were able to calculate yields.

### Using Machine Learning to Understand QSPRs for Biobased Polymer Design

The development of performance-advantaged biobased polymers can be accelerated as the relationship between unique biobased molecular structures and material performance is better understood.^47^ This work developed two approaches to leverage an end-to-end learning algorithm for the exploration of quantitative structure–property relationships.

**Figure 5:**
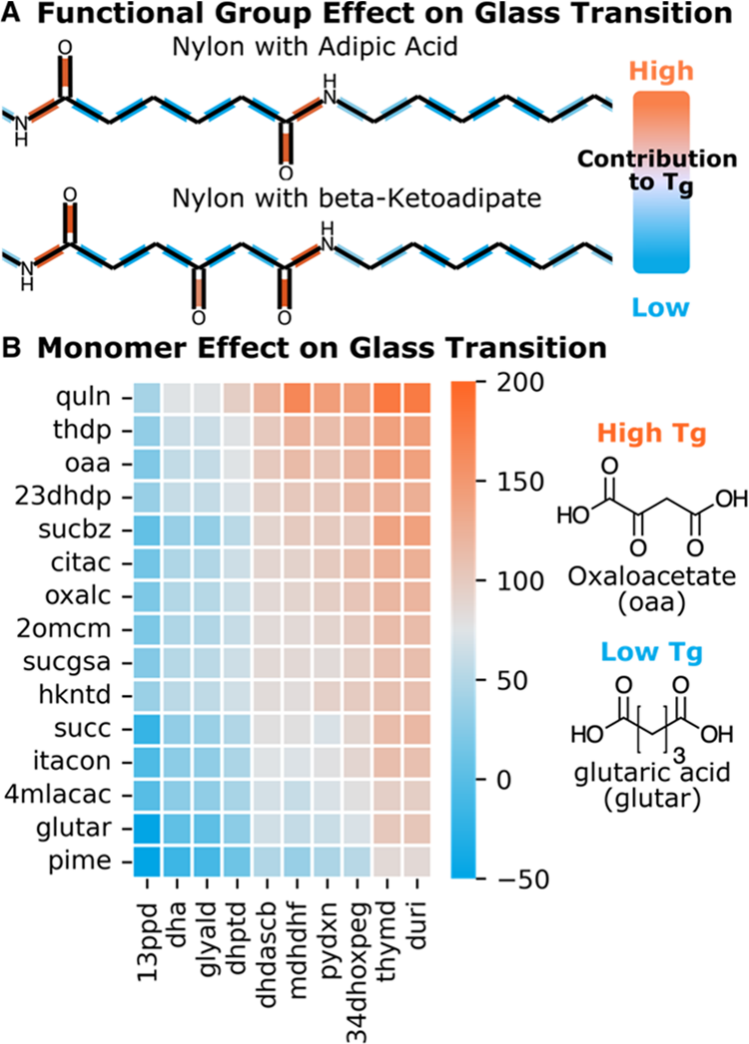
(A) Relative bond contribution to the glass transition temperature for nylon-6,6 and nylon with adipic acid replaced with β-keto adipic acid. (B) Selection of diacid (*y*-axis) and diol (*x*-axis) can be tuned to impact the glass transition. Monomer structures are provided in Figure S7.

The first approach involved correlating individual bond contributions to the polymer properties. This was achieved by pooling each bond vector from the last message-passing block into a single value. The sum of all bonds is then used as the prediction value, which forces the algorithm to assign a relative contribution for all bonds, and has been exemplified using the two nylon polymers shown in Figure 5A.^18^ The β-ketone in β-ketoadipate is known to increase *T*_g_ relative to that in the adipic acid–based polyamide. Molecular dynamics has shown that the experimentally verified increase in *T*_g_ is attributed to decreased rotational freedom around the β-ketone, which increases the rigidity of the carbon polymer backbone.^48,49^ While PolyID cannot provide the same mechanistic insights as molecular dynamics, the increase in *T*_g_ is appropriately assigned to the β-ketone, and thus, some structure–property relationship can be inferred by the high-throughput prediction algorithm. The second approach aimed to inform biobased polymer design by understanding how individual monomers independently affect material properties. Heat maps based on the predicted property, as shown in Figure 5B, can be used to identify trends in monomer contribution and aid in the selection of monomers with the desired performance. By studying bond contribution to properties and monomer impact on materials, the ability to understand QSPRs for new biobased materials can be improved and used to enhance the performance of these biobased materials.

## Discussion

The aim of this work is to accelerate the transition to polymers derived from sustainable feedstocks by applying high-throughput property prediction to identify performance-advantaged biobased polymers. Experimentally screening the 1.4 × 10^6^ biobased polymers identified from four biomolecule databases is not feasible. High-throughput computational tools for predicting material performance, such as PolyID, are thus necessary to hasten the transition to biobased polymers. Figure 4F identifies two clear gaps in computational analyses that, if filled, can improve the screening of performance-advantaged biobased polymers. (1) The number of biobased polymers that fall within the domain of validity needs to be increased by expanding the size and chemical diversity of training databases so there is coverage in chemical space relevant to biobased polymers. (2) The yields of biobased monomers that can be determined from constraint-based metabolic models need to be increased by expanding the pathways incorporated into these models.

The design space can be further expanded if additional polymer chemistries, automated network generation, synthesis planning tools, and/or reinforcement learning approaches are applied to available bioaccessible molecules.^50−53^ Here, an initial attempt toward coupling constraint-based metabolic modeling with machine learning-based property prediction was pursued to identify polymers with performance advantages and carbon-efficient production routes. However, increased coverage of bioaccessible chemical motifs in training sets and expansion of modeled metabolic pathways will be needed before the combination of these computational methods can play a significant role in the development of biobased polymers. Further, maximum theoretical yields for biological transformations are rarely achieved experimentally, and technoeconomic models for these products will need to account for lower yields and separations costs. In practice, downstream chemical modification of bioderived products is common. Incorporating retrosynthesis methods where bio- and chemo-transformations are combined would further expand the available chemical domain and is an opportunity to link property prediction to an even broader set of carbon-efficient production pathways.^53^

Developing high-throughput property prediction tools that are both accurate and interpretable is challenging for chemically complex systems, such as polymers. This work demonstrated that machine learning methods applied to polymers can achieve greater accuracy when the depth of the network architecture is increased and when the structural representation of the polymers moves beyond simple repeat units. This greater accuracy is demonstrated in Table S11 which compares the performance of the message passage neural network with other reported embedding and prediction models using the same training and test set. The lack of benchmark data sets in polymer informatics makes direct comparison challenging across models and remains a challenge for the field. With precise control over polymer topology, future end-to-end learning on polymers could incorporate molecular weight or sequence (*e.g*., stereo-, regio-) controlled polymers while being coupled with coarse-graining type methods, such as BigSMILES, to enable computationally tractable machine learning on macromolecular architectures (e.g., block copolymers).^54,55^ Design principles for biobased polymers are nascent compared to their petro-based counterparts, which is in part due to the vast structural diversity available through bio- and chemo-catalytic transformations. Quantitatively determining the direction and magnitude of performance changes due to bioaccessible structural modifications, such as adding hydroxy and methoxy groups to isophthalic acid, in a high-throughput method can aid in developing biobased alternatives to petrochemical incumbent materials. In this work, we have shown how to build high-throughput machine learning tools that can elucidate quantitative structure–function relationships at the bond or monomer level. Advanced machine-learning algorithms, such as variational autoencoders or self-attention models, could further increase the interpretability of latent-space embedding representations and help to develop design principles for biobased polymers.^56,57^

## Conclusions

In this work, a new, high-throughput polymer property prediction tool, PolyID, was developed using an end-to-end learning approach. The prediction accuracy of the multioutput, message-passing neural network was improved by coupling neural network architecture design and polymer structure representation. The tool was validated through in-house experimental synthesis of 10 polyesters and 12 polyamides that were not contained in the training set. The discovery of a biobased, performance-advantaged PET analogue was achieved by screening >22,000 polyester candidates for improved thermal and barrier performance using PolyID. The improved thermal performance of the PET analogue was confirmed experimentally. By using high-throughput property prediction tools to identify materials that have enhanced performance and are accessible from biobased or waste-based substrates, the transition to polymers derived from renewable feedstocks can occur more quickly.

## Methods

### Preparation of the Labeled Database

A labeled database was constructed by aggregating existing polymer databases^58−61^ and literature reports resulting in 1,791 unique polymer structures, Table S12, across the 5 polymer classes, Figure S7, and 8 polymer properties. Data, monomer structures, and polymer structures generated from the structure building code that were curated from literature reports are provided in Table S13 in machine-readable format. The database was created by compiling monomers represented as SMILES with one or more of the following polymer properties: glass transition temperature (*T*_g_), melt temperature (*T*_M_), density (ρ), modulus (*E*), and the permeability of O_2_, N_2_, CO_2_, and H_2_O (*P*_M-O_2__, *P*_M-N_2__, *P*_M-CO_2__, *P*_M-H_2_O_). Data statistics are provided in Table S4. During data aggregation, monomer SMILES strings were matched with the corresponding polymer properties, and entries with multiple values for the same monomer set and property were averaged. Data quality was ensured for each monomer set through manual data review to ensure correctness of monomer structure and property value and by verifying monomers would undergo the desired chemistry using automated polymer reaction schemes.^62^ From each monomer set, a polymer structure was generated using known polymer chemistries. The polymer structure and corresponding vector of property values were used for training.

### Polymer Structure Building Algorithm

The polymer structure building code, dubbed “monomers to polymers” (m2p), reacts monomers together to produce a polymer chain that are all represented as SMILES. To generate polymer SMILES from monomer SMILES, 5 polymer reaction chemistries were encoded in RDKit’s reaction SMARTS, which are provided in Figure S8. To initiate the polymerization reaction, two monomers that are known to undergo the specified chemistry are probabilistically selected based on the molar ratios provided. If no molar ratios are provided, equal molar is assumed. This creates a polymer chain with a degree of polymerization of 2. A new monomer is selected using the same probabilistic selection process and is reacted with the growing polymer chain. This process continues until the specified degree of polymerization is reached. Each monomer is inserted into the growing chain with random regio-selectivity. The structure building algorithm can build polymer structures with varying degrees of polymerization, molar ratios, and stereochemistry based on user input. The code is also able to generate replicate structures based on the same monomer set wherein monomer order within the polymer chain varies due to random sampling based on the monomer molar ratios. m2p and an example jupyter notebooks are available at www.github.com/NREL/m2p. The python package may also be installed via pypi: *pip install m2p*. In this work, a single polymer chain was generated for training, and 7 replicate polymers were generated when making new predictions. MAE did not improve by increasing the number of replicate polymers for training, and the prediction variability was sufficiently reduced when 7 or more replicate structures were used in the prediction of new polymers.

### Splitting Training, Validation, and Test Sets

Twenty percent of the labeled database was set aside as a test set. The remaining data was segregated using a 10-fold cross-validation strategy using scikit learn, which resulted in 10 trained models for prediction.^63^ Prediction uncertainties for new polymer structures are calculated by taking the mean of the predictions from all 10 models. Error bars for predicted values indicate the standard deviation for predictions made by the 10 trained models produced from the 10-fold cross-validation. Training, validation, and test sets were stratified across polymer classes to ensure splits and performance were not biased toward a specific polymer class. Data values were scaled by using the robust scaler from Scikit-Learn.

### Preparation of the Prediction Database

To source a database of biobased monomers for prediction, four metabolic analyte databases, MetaCyc, MINE, KEGG, and BiGG, were used to identify compounds that are bifunctional for thermoplastics condensation polymerization, which have a molecular weight below 300 Da for processability, and which only contain only CHNO atoms.^32−35^ After monomer screening, the number of fully biobased polyesters, polyamides, and polycarbonates was determined for each database, Figure 4A. The total number of polymers, after considering database overlap, was 1.4 × 10^6^, Table S9.

### Graph Neural Network Architecture, Training, and Hyperparameter Optimization

The graph neural network used in this work is based on previously developed message-passing neural networks and used the neural fingerprints python library.^64^Figure S9 provides a diagram of the PolyID pipeline and the PolyID network architecture. The loss function used the mean absolute error across all 8 material properties, and missing values were masked. Each model was trained using gradient descent by iterating over the data set for 1000 epochs with the ADAM optimizer, which was sufficient for the validation loss to achieve an asymptotic value. Exemplary loss and validation loss plots as a function of epoch number are provided in Figure S10. Hyperparameter optimization was performed for network design parameters and polymer structure: batch size, initial learning rate, decay rate, atom and bond feature vector length, degree of polymerization, and number of message-passing layers. Parameter ranges and optimal values are provided in Table S1, and test set loss vs parameters’ values is provided in Table S3. Optimal values were selected based on test set performance wherein test loss no longer improved by increasing the parameter or an optimal value for the parameter was found.

### Domain of Validity

A domain of validity method was established to measure the structural similarity between polymers in the training and prediction sets. This was achieved using Morgan fingerprints, available through RDKit, to generate hashes associated with chemical substructures in a polymer structure.^27^ All hashes within the training data set were generated, and hashes were then generated for the polymer structures that were being predicted. The number of hashes in the prediction structure that were not found in the training database were summed. The radius for molecular fingerprinting was 2. For a polymer to be considered within the domain of validity, a threshold of ≤6 was selected for the number of hashes not in the training set.

### Polyamide Synthesis

Stock solutions of 1.5 M imidazole, 1.5 M triphenylphosphite (TPP), 0.25 M diacid, and 0.25 M diamine were prepared for synthesis reactions. All solutions used dimethylformamide (DMF) as a solvent and were sonicated as necessary to dissolve constituents.^65^ Equal volumes of TPP, imidazole, diamine, and diacid were added to a glass reactor vessel and placed on a stir plate for 24 h. After reaction, 4–6× the reaction volume of acetone was used to precipitate the contents. To purify the polymer, the precipitate was vacuum-filtered and rinsed with water and acetone 5× each, while crushing the powder further between each rinse. The powder was then dried in a vacuum oven overnight at 60 °C. The acetone/water rinse and vacuum drying were repeated 3×. Alternatively, if the precipitate was water-soluble, the contents were placed in a 50 mL centrifuge tube with ∼40–50 mL acetone. The contents were centrifuged for 3 min at 10,000 rpm, and the acetone was decanted. Fresh acetone was added, and the contents were stirred and allowed to centrifuge. The precipitate was centrifuged for a total of 3×. The contents were placed in the vacuum oven overnight at 60 °C. The centrifuge and vacuum oven process were repeated 3×. Polymer samples were then prepared for analysis.

### Polyester synthesis

Prior to polyester synthesis, each diacid of interest was converted to the corresponding dimethyl ester via reflux in methanol with sulfuric acid as a catalyst. Post esterification, the dimethyl ester was purified via silica gel chromatography. All polyesters used the standard synthesis protocol except in the case of poly(ethylene 5-carboxyvanillate) wherein the standard and extended synthesis protocols were performed.

#### Standard Synthesis

Polyesters were synthesized by adding a dimethyl ester, a diol, and a catalyst (titanium(IV) butoxide/antimony(III) oxide) at a 1:1.1:0.025 molar ratio to a round-bottom flask, respectively. A short-neck distillation apparatus was fixed to the flask, and the reactants were heated to 140 °C under nitrogen and held for at least 12 h. Still under nitrogen, the temperature was increased to 220 °C for 4 h. Finally, the pressure was reduced to ∼50 mTorr while remaining at 220 °C for an additional 4 h. The reaction mixture was cooled to room temperature for purification. The polymer was purified by initially solubilizing the material in a minimal amount of chloroform, followed by reprecipitation in excess cold methanol. The solution was stored at 4 °C overnight to encourage further precipitation. The precipitated polymer was vacuum-filtered, and the purification process was repeated once more.

#### Extended Synthesis

The dimethyl ester of 5-carboxy vanillic acid was charged into a round-bottom flask with a stir bar, ethylene glycol, and titanium(IV) butoxide at a 1:1:0.05 molar ratio. A short-neck distillation apparatus was fixed to the flask, and the reactants were heated under nitrogen to 150 °C for 24 h. Still under nitrogen, the temperature was increased to 200 °C for 22 h. The product was brought to room temperature overnight and then heated again at 200 °C for 7 h under vacuum (∼10 mTorr). The reaction mixture was analyzed without purification due to the insolubility of the material.

### Polymer Characterization

Polymer thermal analysis was performed on a TA Discovery 25 Digital Scanning Calorimeter (DSC). Samples were first annealed with the instrument through an initial temperature cycle, and then thermal properties, glass transition temperature, and melt temperature were extracted from the second cycle thermograms. A scan rate of 10 °C/min was used, and TA Universal Analysis software was used to extract the property values from the generated thermograms. Prior to DSC analysis, samples were analyzed via thermogravimetric analysis (TGA), TA Q5500 TGA, to verify that residual solvent had been removed and determine degradation temperatures. NMR was also used to determine the polymer molecule weight via end-group analysis. Specifically, the polymers were dissolved in d-CDCl_3_ or d-TFA and subject to analysis on a Bruker Advance III HD 400 MHz NMR spectrometer. A relaxation time of 30 s across 16 scans was used to collect ^1^H NMR spectra. A Bruker Advance III HD 400 MHz NMR spectrometer was used for structural identification.

### Metabolic Modeling

The cobrapy python package was used to calculate maximum theoretical yields using the iJO1366 *E. coli* model, the *S. cerevisiae* consensus model v8.4, the *P. putida* model, and an *Arabidopsis* model.^38−40,46^ Metabolite yields were calculated by adding a demand reaction for each metabolite contained in the model and optimizing the flux through that reaction while maintaining ATP constraints defined by each model. The carbon yield was defined as the carbon flux for the metabolite of interest divided by the total carbon flux in. For *S. cerevisiae*, *P. putida*, and *E. coli* the primary carbon source was glucose, with some products also involving uptake of CO_2_. For *Arabidopsis*, the carbon source was CO_2_. All metabolites consisting of CHNO, with at least two carbon atoms, a molecular weight less than 300 Da, and a carbon yield greater than 10% were considered for further yield analysis.

## Data Availability

Polymer structure building code is available at www.github.com/NREL/m2p. The code for building the message-passing neural network is available at www.github.com/NREL/nfp. The code for building the message-passing neural network is available at www.github.com/NREL/polyid. Pypi packages nfp, m2p, and polyid are also available for installation. A web-based interface that serves the models and makes predictions is available at https://polyid.nrel.gov/. A set of examples for generating and training a message-passing neural network, generating polymer structures, predicting with a trained neural network, determining a domain of validity, and generating hierarchical fingerprints can be found at https://github.com/NREL/polyID/tree/master/examples.

## Supporting Information Available

Additional data and results supporting the findings with this manuscript (PDF)
Database of polymer properties that can be used for training (XLSX)
Data table of metabolites that are reachable through known metabolic pathways along with associated calculated yields and structural functionality (XLSX)
