# Supplementary material for: PolyID: Artificial Intelligence for Discovering Performance-Advantaged and Sustainable Polymers
Source: Macromolecules. 2023 Oct 19;56(21):8547–57. doi: 10.1021/acs.macromol.3c00994 (PMC10653284; doi:10.1021/acs.macromol.3c00994)
Supplement: Supplementary file 1 — ma3c00994_si_001.pdf [file ma3c00994_si_001.pdf]

Supporting Information for

PolyID: Artificial Intelligence for Discovering Performance-Advantaged and Sustainable Polymers

A. Nolan Wilson,[1,4,7,*] Peter C. St John,[1,4,8] Daniela H. Marin,[1,5] Caroline B. Hoyt,[1,6] Erik G. Rognerud,[1] Mark R. Nimlos,[1] Robin M. Cywar,[1] Nicholas A. Rorrer,[1] Kevin M. Shebek,[1,2,3] Linda J. Broadbelt,[4] Gregg T. Beckham,[1] Michael F. Crowley[1]

[1] Renewable Resources and Enabling Sciences Center, National Renewable Energy Laboratory, 15013 Denver W Pkwy, Golden, CO 80401, USA
[2] Department of Chemical and Biological Engineering and Center for Synthetic Biology, Northwestern University, Evanston, IL 60208, USA
[3] Chemistry of Life Processes Institute, Northwestern University, Evanston, IL 60208, USA
[4] These authors contributed equally.
*Corresponding author: nolan.wilson@exxonmobil.com

Table of Contents

Supplementary Tables
Table S1 – Hyperparameter results.
Table S2 – Single vs. Multi-task predictions.
Table S3 – Hyperparameter loss and error data.
Table S4 – Database statistics and prediction performance.
Table S5 – Domain of validity metric.
Table S6 – Impact of data on accuracy.
Table S7 – Experimental data for validating model performance.
Table S8 – Theoretical yields for metabolites in metabolic model.
Table S9 – Accessible bio-based polymers.
Table S10 – Theoretical yields and predicted polymer properties.
Table S11 – Comparing Model Performance
Table S12 – Database composition.
Table S13 – Database of polymers and properties from literature reports

Supplementary Figures
Figure S1 – Latent space embedding.
Figure S2 – Hyperparameter optimization for polymer property prediction using message passing neural networks.
Figure S3 – Test set loss as a function of network depth and polymer size
Figure S4 – NMR of selected synthesized polymers.
Figure S5 – Polymer structures of performance-advantaged PET replacements.
Figure S6 – Poly(ethylene 5-carboxyvanillate) analysis
Figure S7 – Select diols and diacids from bio-based monomer database.
Figure S8 – *In silico* polymerization scheme.
Figure S9 – PolyID pipeline and graph neural network architecture.
Figure S10 – Training loss.

Figure S1 – Latent space embedding.

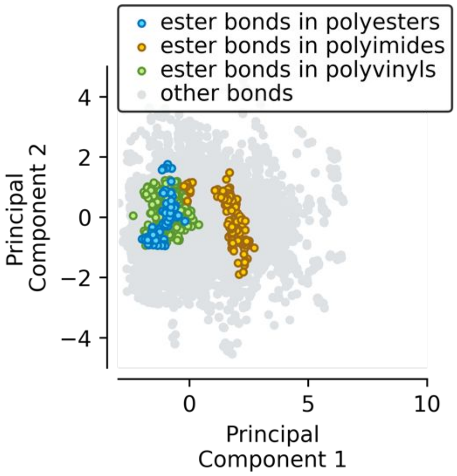

The bond latent space embedding is shown by plotting the two principal components from the bond vectors. The plot shows ester bonds spatially differentiate themselves in latent space based on the polymer type after multiple message-passing layers.

Figure S2 – Hyperparameter optimization for polymer property prediction using message passing neural networks.

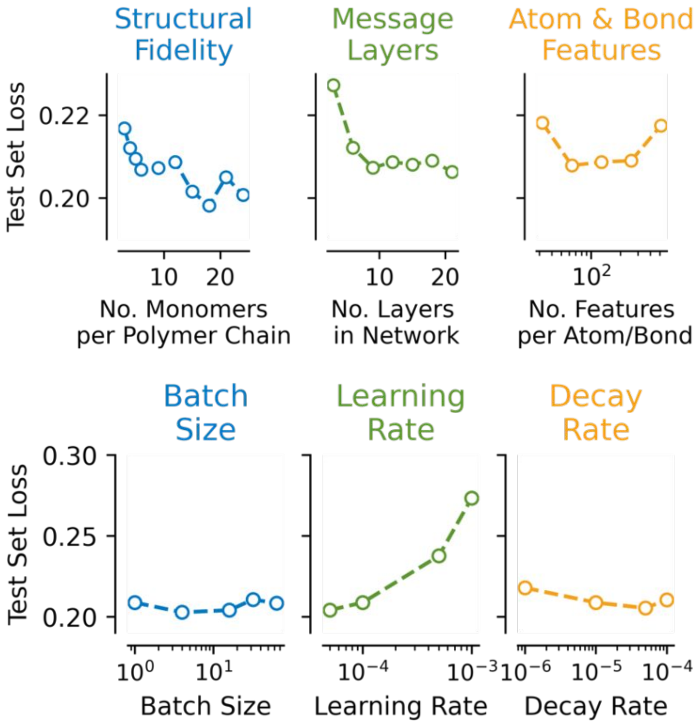

Effect of polymer structure, graph network topology, and training parameters on test set loss for all 8 predicted polymer properties.

The approach to hyperparameter optimization here was twofold: (1) to provide insights into the relationship between network design (*e.g.*, number of message passing layers, size of atom and bond feature vectors) and polymer structural representations (*e.g.*, monomers in each polymer chain), and (2) to select a set of hyperparameters that perform reasonably well. While exhaustive grid search or advanced hyperparameter optimization techniques could potentially identify a more optimal set of hyperparameters, the approach taken here balances scientific understanding and computational expense.

In **Figures 2A-C** and **Figure S2** each datapoint represents an independent message passing neural network trained with different hyperparameters. To train each independent network, a standard 10-fold cross-validation was used and a single hyperparameter value was changed while holding all other hyperparameters constant. Each of the 10 k-fold models were evaluated using a hold-out "test" set and the average error was calculated across the 10 k-folds. **Table S1** provides the ranges for each independently varied hyperparameter, the constant values used for the other hyperparameter variables when that variable was not the varied hyperparameter, and the determined optimal value. **Figure S2** shows the results of the test set loss, which represents the aggregate error across all 8 predicted properties and 10 k-fold models. **Figures 2A-C** shows the melt temperature mean absolute error to provide a more interpretable and exemplary version of the results.

Figure S3 - Test set loss as a function of network depth and polymer size.

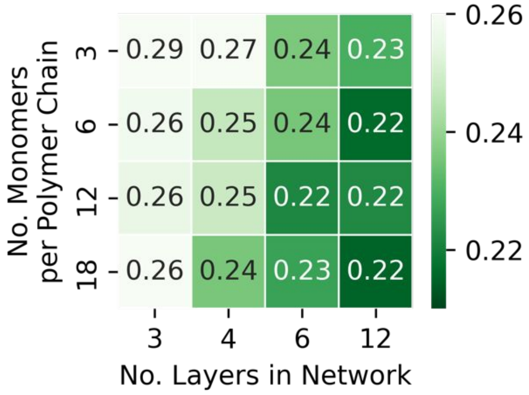

Test set loss (lower is better) as a function of the number of monomers in a polymer chain and the number of message passing layers in the network. **Figure S3** shows the benefit of increasing network size when increasing polymer size holds true for unseen data (*i.e.*, test set). The effect is also presented in **Figure 2D** for the validation set loss. To generate this data, all other hyperparameters remained the same. The number of monomers per polymer chain and the depth of the network were varied and 16 different models were trained and evaluated using a 80/20 train/test split. Each training set used 10-fold cross validation during training.

Table S1 – Hyperparameter results. Ranges used and optimal values determined in hyperparameter optimization scheme of message passing neural network for making predictions on polymer structures. The constant values are the values used for the other parameters while a single parameter was being varied within the range of values.

Design Parameter	Range of Values	Optimal Value	Constant Value
Batch Size	1 – 64	1	1
Learning Rate	5×10^{-5} – 5×10^{-3}	1×10^{-4}	1×10^{-4}
Decay Rate	1×10^{-6} – 1×10^{-4}	1×10^{-5}	1×10^{-5}
Atom & Bond Feature Vector Length	32 – 512	128	128
Number of Messages	3 – 21	12	12
Degree of Polymerization	3 – 24	18	12

Table S2 - Single vs. Multi-task predictions. Ranges used and optimal values determined in hyperparameter optimization scheme of message passing neural network for making predictions on polymer structures.

Properties used in Training	T_g Test Set MAE (°C)	T_M Test Set MAE (°C)	ρ Test Set MAE (g mL^{-1})
T_g	19.2	-	-
T_g, T_M	19.2	26.9	-
T_g, T_M, ρ	**19.1**	28.4	**0.04**
T_g, T_M, ρ, E, $P_{M\text{-}O2}$, $P_{M\text{-}N2}$, $P_{M\text{-}CO2}$, $P_{M\text{-}H2O}$	19.8	**25.2**	0.05

To compare prediction accuracy as a function of the number of properties being predicted by a network, four models were trained using the same hyperparameters. Only the properties used in the training task were varied. In the first model only glass transition was used. In the second model glass transition and melt temperature were used. In the third model glass transition, melt temperature, and density were used. In the fourth model all 8 properties were used. From **Table S2**, no consistent performance improvement for multi-task learning is observed. Others have found more substantial improvements.[1,2] The difference in findings may be due to network architecture, polymer structure representation, data set composition, or data set size. Additional benchmarking studies will be needed to determine benefits of multi-task learning.

Table S3 – Hyperparameter loss and error data. The mean absolute error as shown in **Figure 2C** and the test set loss data for **Figure S2**.

No. monomers per polymer chain	T_M MAE (°C)	atom feature vector size	T_M MAE (°C)	num of message layers	T_M MAE (°C)
3	27.9	32	28.4	3	31.5
4	27.1	64	26.8	6	28.2
5	26.9	128	26.4	9	26.9
6	27.3	256	27.3	12	26.4
9	24.4	512	29.8	15	26.2
12	26.4			18	25.8
15	26.8			21	25.6
18	25.1				
21	25.2				
24	25.2				

no. monomers per polymer chain	test set loss	atom feature vector size	test set loss	num of message layers	test set loss
3	0.217	32	0.218	3	0.227
4	0.212	64	0.208	6	0.212
5	0.209	128	0.209	9	0.207
6	0.207	256	0.209	12	0.209
9	0.207	512	0.217	15	0.208
12	0.209			18	0.209
15	0.202			21	0.206
18	0.198				
21	0.205				
24	0.201				

batch size	test set loss	learning rate	test set loss	decay rate	test set loss
1	0.209	0.00005	0.204	0.000001	0.218
4	0.203	0.0001	0.209	0.00001	0.209
16	0.204	0.0005	0.238	0.00005	0.205
32	0.211	0.001	0.273	0.0001	0.210
64	0.208				

Table S4A – Database statistics and prediction performance for the full database. Training database statistics, mean absolute errors for 10-fold validation set, and mean absolute errors 20% hold-out test set. The database used for training and evaluating the model that produced the data in this table included data in *SI_Table-of-polymer-properties.csv* and external databases that could not be reproduced due to copyright. Details of the full database are provided in the Methods section of the main text.

Parameter	Database Set		Validation Sets	Test Set
	Data Range	Data Size	MAE	MAE
Glass Transition (°C)	-40 – 330	1,321	20.9	19.8
Density (g mL^{-1})	0.91 – 1.42	395	0.058	0.05
Permeability (Barrer)				
O_2	$10^{-1.1}$ – $10^{2.1}$	338	$10^{0.2}$	$10^{-0.1}$
CO_2	$10^{-1.1}$ – $10^{2.6}$	284	$10^{0.8}$	$10^{0.4}$
N_2	$10^{-1.7}$ – $10^{1.6}$	317	$10^{-0.3}$	$10^{-0.8}$
H_2O	$10^{0.5}$ – $10^{3.4}$	27	$10^{2.6}$	$10^{2.4}$
Melt Temperature (°C)	-27 – 333	390	29.4	25.2
Modulus (MPa)	77 – 3300	423	430	340

Table S4B – Database statistics and prediction performance for published database. Training database statistics, mean absolute errors for 10-fold validation set, and mean absolute errors 20% hold-out test set. The database used for training and evaluating the model that produced the data in this table only included data in *SI_Table-of-polymer-properties.csv*.

Parameter	Database Set		Validation Sets	Test Set
	Data Range	Data Size	MAE	MAE
Glass Transition (°C)	-39 – 330	807	22.1	20.1
Density (g mL^{-1})	0.92 – 1.42	217	0.05	0.05
Permeability (Barrer)				
O_2	$10^{-1.0}$ – $10^{2.1}$	257	$10^{0.2}$	$10^{0.3}$
CO_2	$10^{-1.0}$ – $10^{2.6}$	205	$10^{0.8}$	$10^{0.8}$
N_2	$10^{-1.7}$ – $10^{1.6}$	251	$10^{-0.4}$	$10^{-0.6}$
H_2O	ND	0	ND	ND
Melt Temperature (°C)	28 – 333	46	41	57.5
Modulus (MPa)	77 – 3300	348	366	430

Table S5 – Domain of validity metric. Table containing mean absolute error for glass transition temperature values as a function of the number of substructures for the prediction structures that are outside of the substructures found in the training set. Substructures were generated using Rdkit's Morgan fingerprints method with a radius equal to two. As the number of substructures outside the training set decreases, the mean absolute error improves.

Substructures outside train	T_g MAE (°C)
0	16
1	21
2	20
3	22
4	25
5	29
6	30
7	45
8	37
9	30
10	55
11	38
12	35
13	26
14	67
15	56

Table S6 – Impact of data on accuracy. Table containing mean absolute error for the glass transition temperature for *c,c*-muconic acid-based polymers which used two different models for predicting the values. The first model was parameterized using a training set with no *c,c*-muconic acid-based polymers and the second contained a single instance of a muconic acid-based polymers, poly(1-4 butanediol-co-*c,c*-muconic acid).

Polymer	c,c-muconic acid-based polymers in training set	$T_{g\ observed}$ (°C)	$T_{g\ predicted}$ (°C)	T_g abs. error (°C)
poly(1,4-butanediol-co-*c,c*-mucconic acid)	0	55	0	55
poly(ethylene glycol-co-*c,c* -mucconic acid)	0	60	14	46
poly(1,3-propanediol-co-*c,c* -mucconic acid)	0	47	7	40
poly(1,6 hexanediamine-co-*c,c* -mucconic acid)	0	55	89	34
poly(1,4-butanediol-co-*c,c* -mucconic acid)	1	55	54	1
poly(ethylene glycol-co-*c,c*-mucconic acid)	1	60	55	5
poly(1,3-propanediol-co-*c,c*-mucconic acid)	1	47	57	10
poly(1,6 hexanediamine-co-*c,c*-mucconic acid)	1	55	109	54

Table S7 – Experimental data for validating model performance. Properties of experimentally synthesized bio-accessible polymers and associated predictions for T_g and T_M.

Class	Monomer A	Monomer B	$T_{g\ observed}$ (°C)	$T_{g\ predicted}$ (°C)	$T_{M\ observed}$ (°C)	$T_{M\ predicted}$ (°C)
Polyamide	Adipic Acid	1,6 Hexanediamine	43	47	-	247
	c,c-Muconic Acid	1,6 Hexanediamine	55	81	220	247
	Glutaric Acid	1,4 Butanediamine	54	65	245	268
	Glutaric Acid	1,6 Hexanediamine	62	53	229	232
	Glutaric Acid	4-(2-Aminoethyl)aniline	63	151	-	326
	Pimelic Acid	1,4 Butanediamine	23	54	237	247
	Pimelic Acid	1,6 Hexanediamine	54	55	221	216
	Pimelic Acid	4-(2-Aminoethyl)aniline	103	120	-	298
	Suberic Acid	1,4 Butanediamine	0	50	252	254
	Suberic Acid	1,6 Hexanediamine	65	50	221	232
	Succinic Acid	1,4 Butanediamine	17	80	-	284
	Succinic Acid	1,6 Hexanediamine	-21	57	-	250
Polyester	c,c-Muconic Acid	1,3-Propanediol	47	-2	195	72
	c,c-Muconic Acid	1,4-Butanediol	55	-4	-	69
	c,c-Muconic Acid	Ethylene Glycol	60	1	-	80
	Glutaric Acid	1,3-Propanediol	-51	-40	-	13
	Itaconic Acid	1,3-Propanediol	-24	9	-	57
	Pimelic Acid	1,3-Propanediol	-51	-49	-	34
	Succinic Acid	1,3-Propanediol	-27	-20	48	35
	Succinic Acid	1,4-Butanediol	-31	-38	130	34
	Succinic Acid	1,6-Hexanediol	-40	-46	58	54
	Succinic Acid	Ethylene Glycol	-13	1	105	102

Figure S4 – NMR of selected synthesized polymers.

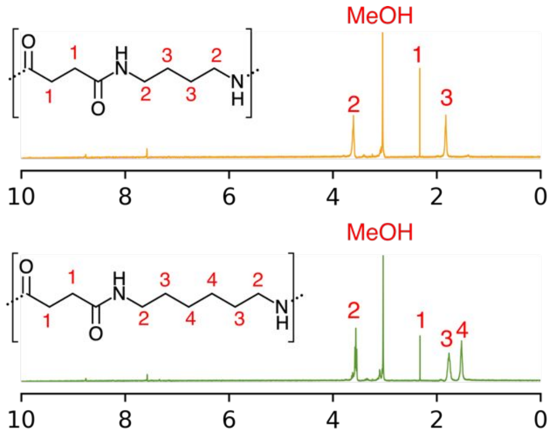

NMR spectra of poly(succinic acid-co-1,4-butanediamine) in yellow (top) and poly(succinic acid-co-1,6-hexamethylenediamine) in green (bottom)

Table S8 – Theoretical yields for metabolites in metabolic model. A table containing values for the raw yield, percent yield, chemical functionality, and monomer class for polymerization for analytes in the four metabolic models.

Table provided in additional attachment SI_Table-of-reachable-metabolites.csv

In **Table S8**, the functionality was determined through a structure-based search for the functionality in each column using Rdkit. Diols, diacids, diamines, hydroxy acids, and amino acids were selected based on the molecular structure. To determine if a monomer would polymerize in polyester, polyamide, or polycarbonate reaction, the following criteria were applied for each monomer type:

Diols: Diols for polymerization contained two hydroxyl groups, and the diols did not contain any acids or primary amines. The hydroxyl groups are aromatic or aliphatic for polycarbonates and for polyesters the hydroxyls are only aliphatic. In the case of polyesters, monomers that contained aromatic hydroxyls were not counted towards the number of hydroxyls in the structure, and the aromatic hydroxyls were not considered for the condensation reactions with acids due to the significant relative reactivity of aliphatic vs. aromatic hydroxyl groups.

Diacids: Diacids for polymerization to make polyesters or polyamides when combined with diols or diamines, respectively, contain two acid groups. The diacids did not contain any aliphatic hydroxyls or primary amines.

Diamines: Diamines for polymerization to make polyamides when combined with diacids contain two primary amine groups. The diamines did not contain aliphatic hydroxyls or acid groups.

Hydroxy acids: Hydroxy acids for polymerization to make polyesters contained one aliphatic hydroxyl and one acid, and the hydroxy acids contained no primary amines. Monomers that contained aromatic hydroxyls were not counted towards the number of hydroxyls in the structure, and the aromatic hydroxyls were not considered for the condensation reactions with acids due to the significant relative reactivity of aliphatic vs. aromatic hydroxyl groups.

Amino acids: Amino acids for polymerization to make polyamides contain one primary amine and one acid. The amino acids contained no aliphatic hydroxyls.

Table S9 – Accessible bio-based polymers. The number of unique polymers that can be generated from each of the following databases: Metacyc, Mines, KEGG, and BiGG.

Database	poly(esters)	poly(amides)	poly(carbonates)	database totals
BiGG	1,326	763	32	2,121
KEGG	22,506	5,727	354	28,587
MINEs	1,531,094	222,972	3,141	1,757,207
MetaCyc	711,599	159,300	1,800	872,699
polymer class totals [a]	1,185,636	201,048	5,566	1,392,250

[a] *Totals for each polymer class account for overlaps between databases and are therefore not the sum of the column.*

Figure S5 – Polymer structures of performance-advantaged PET replacements.

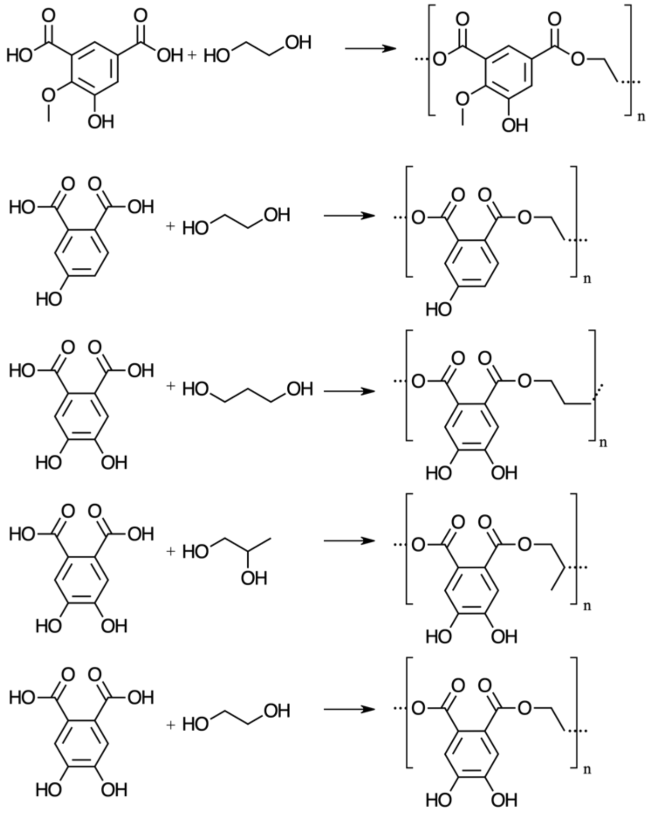

Glass Transition Temperature Predicted (°C)	Glass Transition Temperature Observed (°C)	O_2 Permeability Predicted (Barrer)
106 ± 9	85 - 112	$10^{-0.8 \pm 0.3}$
107 ± 17	ND	$10^{-1.1 \pm 0.3}$
109 ± 13	ND	$10^{-0.8 \pm 0.3}$
140 ± 10	ND	$10^{-1.1 \pm 0.4}$
143 ± 9	ND	$10^{-1.1 \pm 0.4}$

PABP replacements for PET which have been predicted to have a glass transition temperature above 100 °C and have an O_2 permeability equal to or lower than PET. ND indicates the polymer was not synthesized due to limited access to the monomer. The error bars indicate the standard deviation for predictions made by the 10 trained models produced from the 10-fold cross-validation. The only known report for using any of the three identified diacids in polyesters is by Hevus who reported a T_g of 17 °C for poly(1,6-hexanediol 4-hydrophthalate). PolyID was applied to this polymer and predicted a T_g of 31 °C. [3]

Figure S6 – Poly(ethylene 5-carboxyvanillate) analysis – TGA, DSC, FTIR, ^{1}HNMR, ^{13}CNMR, and GPC of dimethyl ester monomer and reaction products from polyester synthesis.

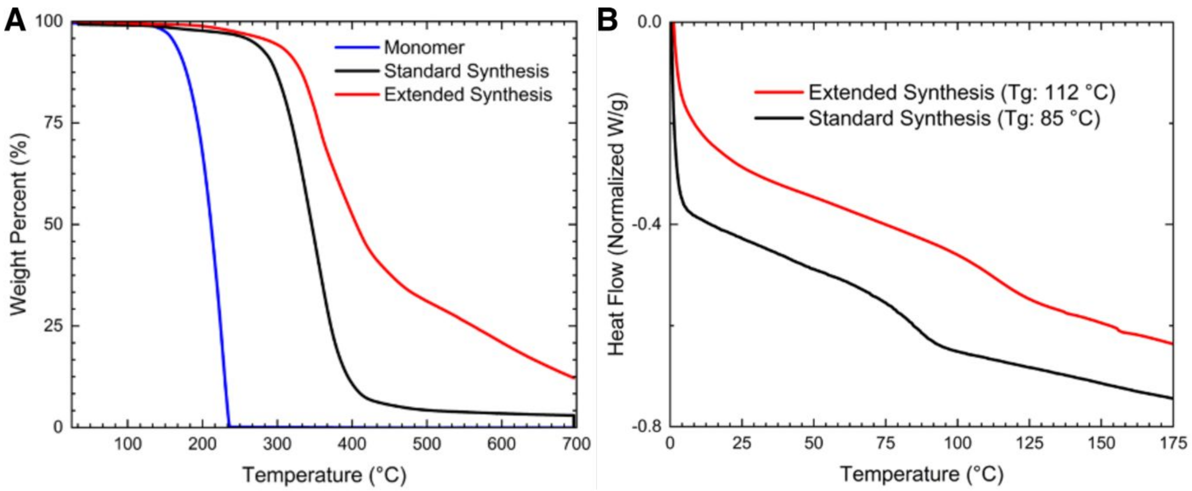

Figure S6A. TGA thermograms of the dimethyl monomer and the two reaction products from differing methods. The observed $T_{d\ 50\%}$ of the monomer, standard synthesis product, and the extended synthesis product are 211 °C, 346 °C, and 405 °C respectively. The increase of ~130 °C in thermal stability provides further evidence of successful polymerization of the polyester. Furthermore, the ~50 °C increase of the extended synthesis suggests higher degrees of conversion with the possibility of crosslinking.

Figure S6B. DSC thermograms of the two reaction products under different synthesis conditions described in the methods section. Polymers were annealed in the DSC Discovery 25 (TA Instruments) on the first cycle to 200 °C at 10 °C/min. Presented here is the second thermal cycle showing that one attempt at a standard polyester synthesis yielded a T_g of 85 °C. An extended synthesis strategy yielded a higher T_g of 112 °C. From this thermal data, it appears that the extended synthesis conditions resulted in higher conversion as indicated by a higher T_g in accordance with prediction from the Fox-Flory equation.

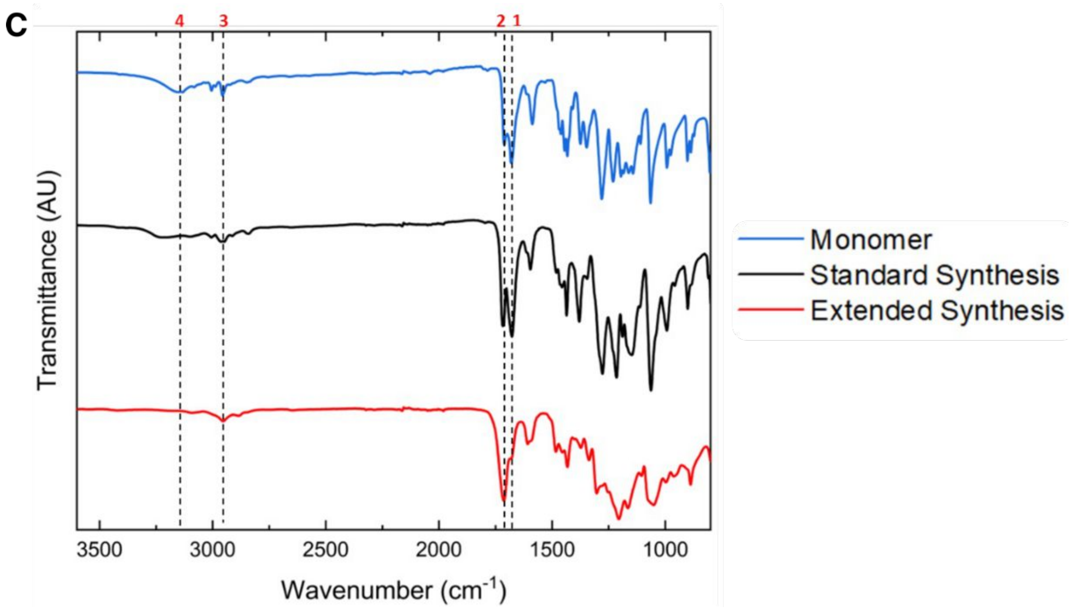

Figure S6C. FTIR transmittance of the dimethyl 5-carboxyvanillate monomer, the reaction product under standard polyester synthesis and the reaction product from an extended synthesis method is shown. The observation of the two carbonyl peaks (**1** and **2**) are indicative of the two carbonyl environments present on the monomer/polymer. As the reaction shifts to longer time, the carbonyl environment shifts to **2** which can be attributed to an averaging of the chemical environments from a higher conversion polymer as the extended synthesis and thus higher molecular weight. The alkane peak (**3**), which is attributed to methoxy on the 5-CVA, is conserved regardless of synthesis strategy. The hydroxy peak (**4**) wanes from the monomer to the standard synthesis as hydrogen bonding is reduced. This peak is practically diminished in the extended synthesis which can be attributed to either higher molecular weights, intra-chain hydrogen bonding becoming more pronounced, or side reactions with the hydroxy group on the 5-CVA during synthesis.

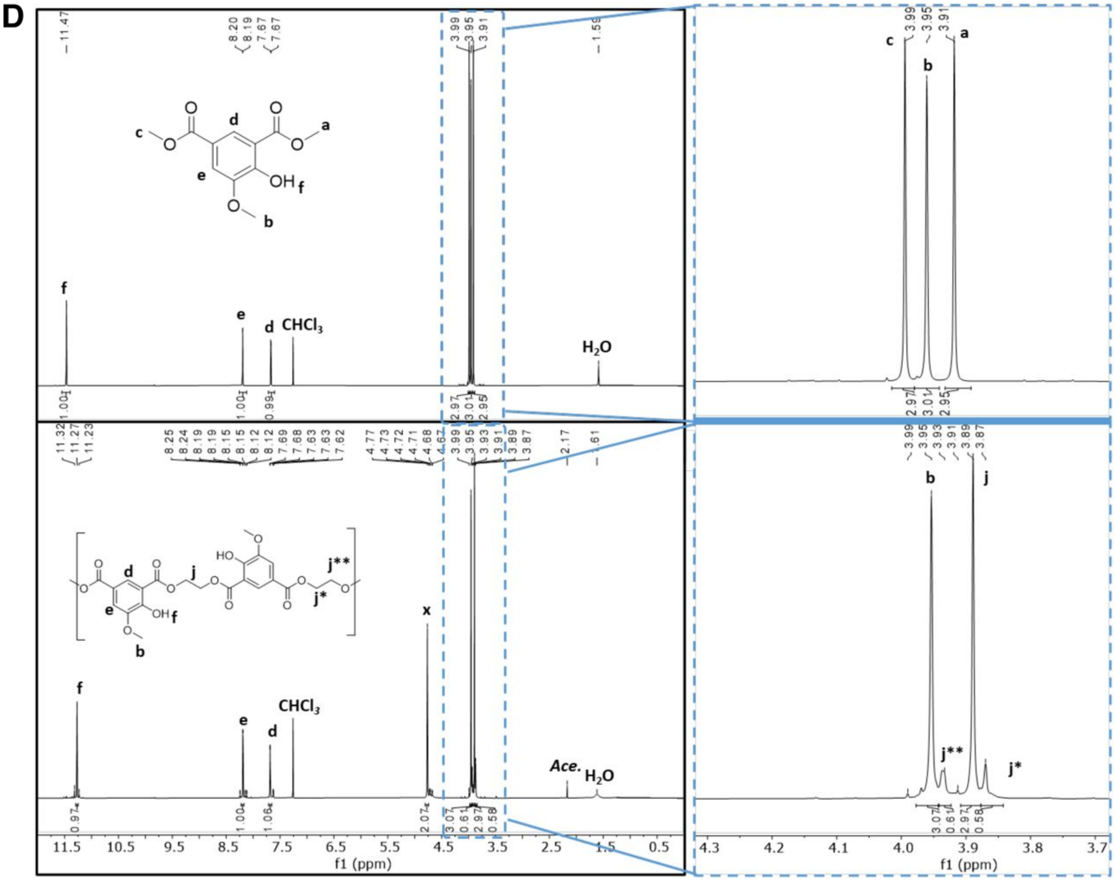

Figure S6D. ^{1}H NMR data collected on a Bruker 400 MHz Spectrometer. **(Top)** Dimethyl 5-carboxyvanillate monomer. (**Bottom)** The reaction product under a standard polyester synthesis. The dimethyl 5-CVA monomer is pure with expected and labeled integrations. The standard synthesis spectra indicates the presences of an ethylene glycol unit between the 5-CVA units, as observed by the presences of peak **j.** As the 5-CVA monomer is non-symmetrical, there is the possibility of different shifts, labeled **j*** and **j****, due to head-to-tail, head-to-head, and tail-to-tail configuration or the possible presence of bis(hydroxy-ethyl) end groups. Additionally, due to the propensity of 1,3 dicarboxylic acid substituted benzenes (e.g. isophthalic acid)[4] to undergo cyclization reaction could lead to the peak of variable integrations, **x**. Importantly, the polymer from the extended synthesis conditions possessed poor solubility in NMR solvents either indicative of side reactions or significantly larger molecular weight. NMR of extended synthesis product not performed due to insolubility.

E

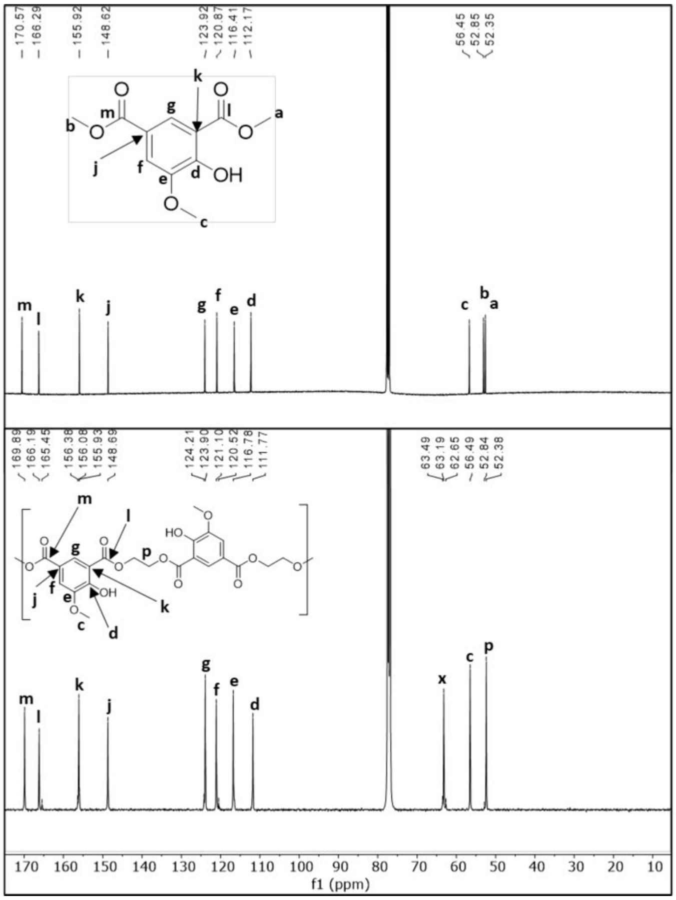

Figure S6E. **^{13}C** NMR data collected on a Bruker 400 MHz Spectrometer to provide structural identification of the monomer (**Top**) and the (standard) reaction product (**Bottom**). The structural identity of the monomer is confirmed in the above spectra. The identification of the 5-carboxy linkage carbon's was confirmed as well. Identification of the ethylene glycol linkages proves to be challenging as an unknown peak appears downfield of the methoxy © and alkane (p) carbon. Similar to the ^{1}H-NMR assignments, this unidentified peak (x) may be attributed to bis(hydroxy-ethyl) end groups or cyclic oligomers. NMR of extended synthesis product not performed due to insolubility.

F

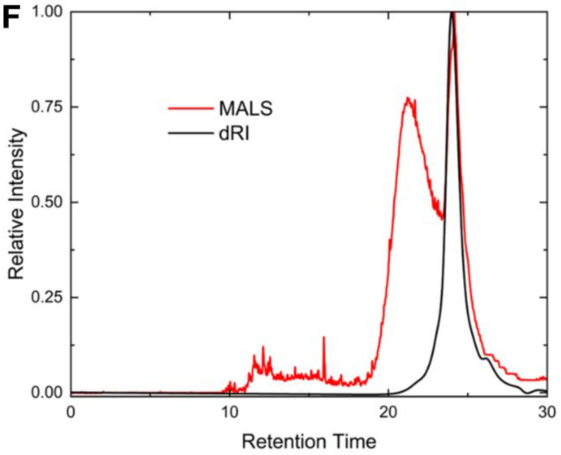

Figure S6F. GPC data of the standard synthesis reaction product collected on an Agilent 1260 Infinity II LC system with a MiniDawn TREOS Multi-Angle Light Scattering (MALS) detector (Wyatt) and an Optilab T-rEX differential Refractive Index (dRI) detector (Wyatt). The polymer product is near the detection limit of the columns and is subject to high uncertainty. Qualitatively, most of the reaction product is low molecular weight polymer with small concentrations of higher molecular weight, as determined by the large intensity of the MALS detector at 22 minutes. Assuming a dn/dc value of 0.2570 (PET), a M_n,M_w, and dispersity were determined to be 608 Da, 1,220 Da, and 2.01. GPC of extended synthesis product not performed due to insolubility.

Table S10 – Theoretical yields and predicted polymer properties. Table containing theoretical yield, predicted glass transition, predicted oxygen permeability, and domain of validity substructure metric for the eight polymers accessible through analytes in the KEGG database for which all of these values could be determined. None of the polymers resulting from these monomer combinations were predicted to be PABP PET replacements.

Monomer A			Monomer B			$T_{g\ predicted}$	P_{O2}	Substructure
Compound	Organism	% Yield	Compound	Organism	% Yield	(°C)	(Barrer)	outside train
Succinic acid	*E. coli*	100	Glycerone	*E. coli*<br>*S. Cerevisiae*	94<br>91	37	$10^{-0.39}$	4
Succinic	*E. coli*	100	(RS)-1,2-Propanediol	*E. coli*	71	19	$10^{-0.10}$	2
Succinate	*E. coli*	100	(R,R)-Butane-2,3-diol	*S. Cerevisiae*	72	44	$10^{0.08}$	3
O-Succinyl-L-homoserine	*E. coli*	99	(RS)-1,2-Propanediol	*E. coli*	71	16	$10^{-0.02}$	6
Glutaric acid	*P. putida*	84	Glycerone	*E. coli*<br>*S. Cerevisiae*	94<br>91	12	$10^{-0.33}$	4
Glutaric acid	*P. putida*	84	(RS)-1,2-Propanediol	*E. coli*	71	-11	$10^{0.14}$	2
Glutaric acid	*P. putida*	84	(R,R)-Butane-2,3-diol	*S. Cerevisiae*	72	18	$10^{0.20}$	3
2-Succinylbenzoate	*E. coli*	88	(R,R)-Butane-2,3-diol	*S. Cerevisiae*	72	56	$10^{0.08}$	6

Figure 7 – Select diols and diacids from bio-based monomer database.

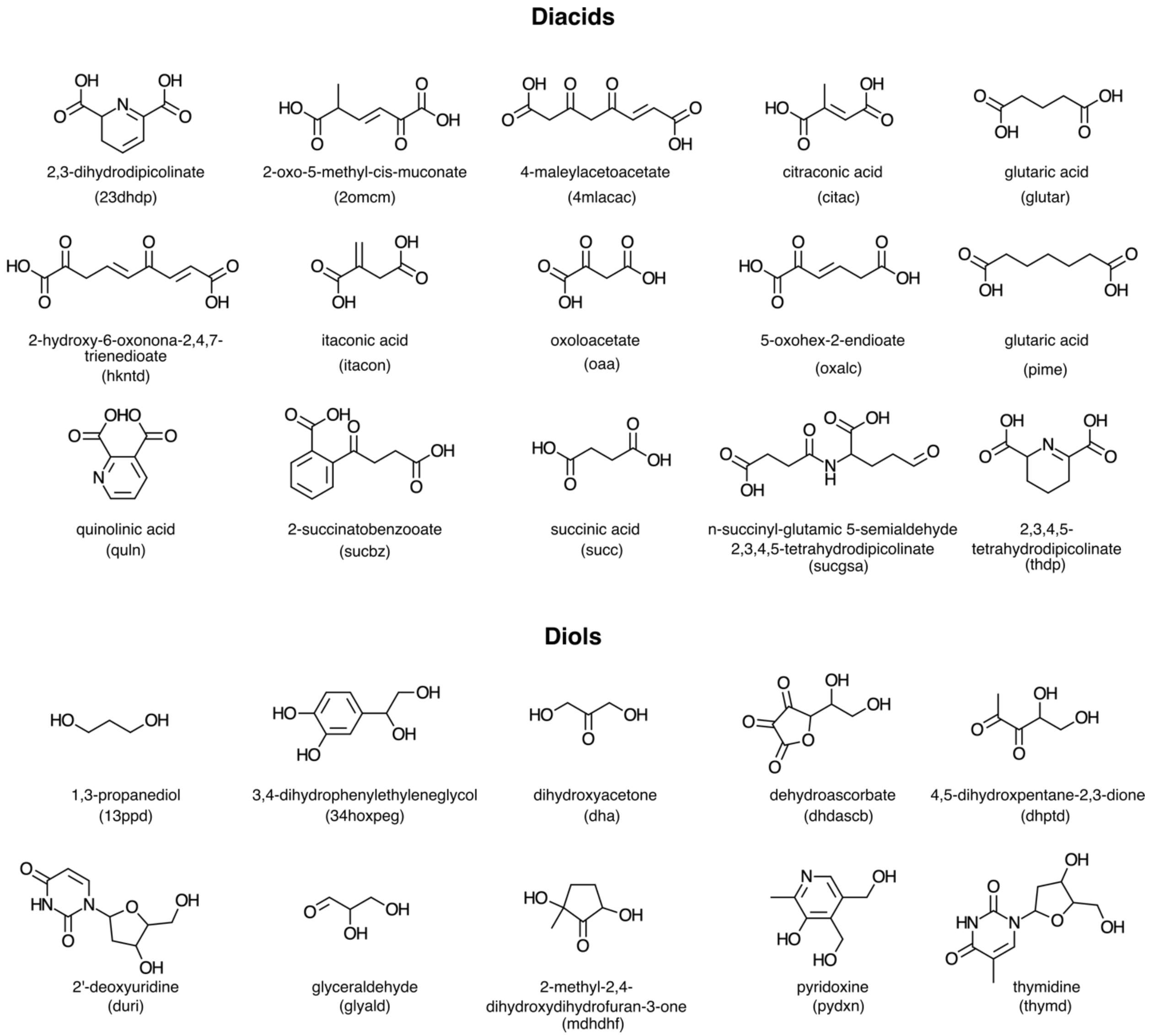

Selection of different comonomers can be used to tune material properties accordingly.

Table S11 – Comparing Model Performance. The table shows the mean absolute error for each predicted property using a hold-out test set. Bold values indicate best performing model for that specific property.

Embedding Method	Message Passing	Hierarchical Fingerprints			Atomistic		
Prediction Method	Neural Network	Neural Network	Gradient Boost	Random Forest	Neural Network	Gradient Boost	Random Forest
Glass Transition (°C)	**19.8**	31.8	25.2	26.2	32.4	25.9	26.9
Melt Temperature (°C)	**25.2**	41.8	39.7	38.2	40.1	33.1	37.1
Density (g mL^{-1})	**0.05**	0.06	0.06	0.06	0.07	0.08	0.07
Modulus (MPa)	**340**	520	490	510	480	500	460
Permeability (Barrer)							
CO_2	**$10^{0.4}$**	$10^{0.9}$	$10^{1.0}$	$10^{0.9}$	$10^{0.8}$	$10^{0.7}$	$10^{0.7}$
H_2O	$10^{2.4}$	$10^{2.5}$	$10^{2.4}$	**$10^{2.3}$**	$10^{2.6}$	$10^{2.6}$	$10^{2.5}$
N_2	**$10^{-0.8}$**	$10^{-0.4}$	$10^{-0.5}$	$10^{-0.6}$	$10^{-0.3}$	$10^{-0.57}$	$10^{-0.6}$
O_2	**$10^{-0.1}$**	$10^{0.3}$	$10^{0.3}$	$10^{0.2}$	$10^{0.2}$	$10^{0.17}$	$10^{0.1}$

We aimed to compare the performance of the message passing neural network developed in this work to state-of-the-art polymer property prediction algorithms. Message passing neural networks combine prediction and embedding tasks into a single end-to-end learning model architecture. Recent reports (2017 to 2022) of polymer property prediction models were identified from the literature. 12 publications containing 37 models were identified that had at least one of the predicted properties in this report.[2,5–15] From this subset, only reports that used a hold-out, "test" set were considered for comparison as test sets most closely simulate prediction of polymer properties that have yet to be experimentally synthesized. Only four reports used a test set and the Kim *et al*. 2018 report, which used hierarchical fingerprints to embed the polymer structure into a latent space, reported the best performance. Hierarchical fingerprints have gained traction in recent years[2,5,6,10,12] and operate by combining fingerprints at different length scales (*e.g.*, atomistic, molecular, and morphological) to create a hand-engineered embedding of the molecular structure. We developed a hierarchical fingerprint embedding based on Kim *et al*. and applied it to the polymer prediction task in this work. Unfortunately, the precise approach used by Kim *et al*. could not be reproduced as no open-source code is available nor are all of the molecular or morphological descriptors listed.

For atomistic scale embedding, we used Morgan fingerprints from RDKit with a radius of 2. This provided a hash that describes unique local environments for different parts of the polymer chain. For molecular and morphological embeddings, Mordred descriptors were used.[16] The mordred descriptor python package provides more than 1,800 descriptors that span molecular and morphological length scales.[17] Atomistic and mordred descriptors were concatenated for each polymer to create the hierarchical fingerprint embedding for each polymer structure. The code to generate our hierarchical fingerprints is provided in the PolyID repository (https://github.com/NREL/polyID/tree/master/examples). Atomistic and hierarchical embeddings were then used to train 3 different machine learning methods from Scikit-learn that resulted in 6 different models.[18] A random search hyperparameter optimization for each model was used to determine optimal parameters. Each model was trained on the same training set used to parameterize the message passing network and used a 10-fold cross-validation.

Each of the 6 models were then compared to the performance of the message passing neural network by evaluating the mean absolute error for each predicted property using the same hold-out test set. From **Table S11**, it is shown that the message passing neural network outperforms on 7 of the 8 prediction tasks. The only property in which the message passing approach did worse was permeability of H_2O, which has significantly lower datapoints relative to the rest of the properties. The lower number of training examples may be the cause of the poorer relative performance. For the other 7 properties, the improved performance can have significant impact on the ability to reduce the number of polymer candidates. For a search space of 1 x 10^6 polymer, a one degree centigrade improvement in the mean absolute error of glass transition temperature can eliminate ~10^5 polymer candidates. By down selecting based on multiple predicted polymer properties, the search space can be reduced to a few hundred that can then be pursued experimentally.

Table S12 – Database composition. Training set database count broken down by polymer class.

Polymer Class	Count
poly(olefins) and poly(acrylates)	524
poly(amides)	370
poly(esters)	339
poly(imides)	524
poly(carbonates)	34

Table S13 – Database of polymers and properties from literature reports. Table containing polymer properties, monomers, and polymer structures that were curated from literature reports. Polymer structures were generated using the monomers-2-polymers code base.

Table provided in additional attachment SI_Table-of-polymer-properties.csv

Figure 8 – *In silico* polymerization scheme.

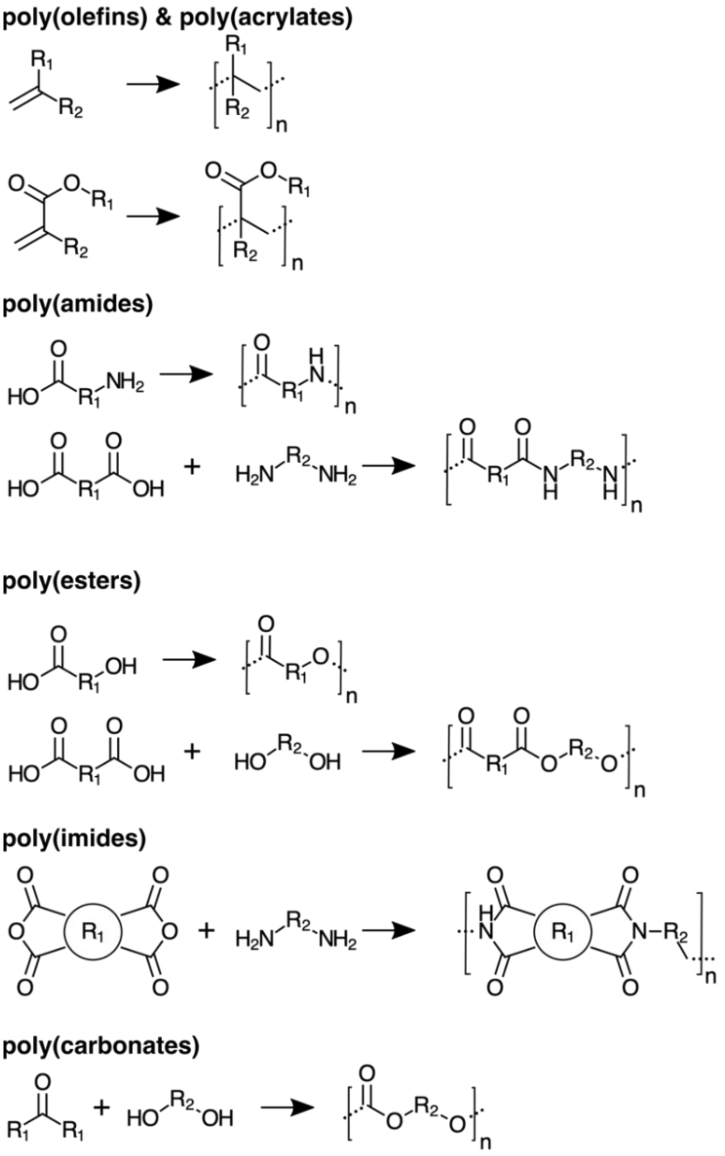

The 5 polymer classes that were incorporated into the *in silico* polymer structure generation scheme.

Figure S9 – PolyID pipeline and graph neural network architecture.

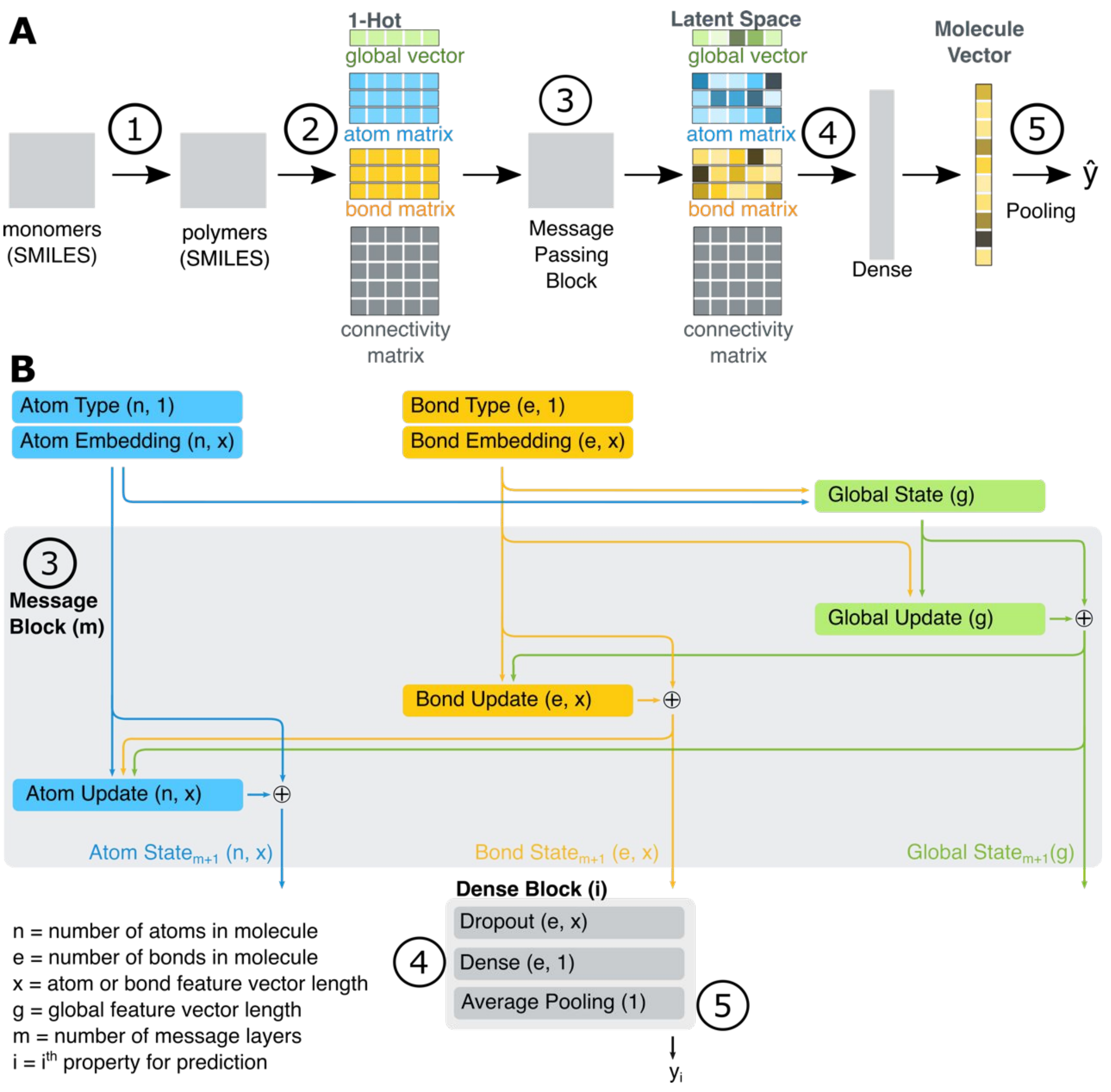

Figure S9 (A) PolyID pipeline and **(B)** PolyID neural network architecture. (1) Monomer SMILES are input and reacted *in silico* to generate polymer SMILES. (2) For each polymer atom and bond feature, matrices are generated by assigning a one-hot encoding based on the atom or bond type (*e.g.*, atom types: carbon, hydrogen, etc. or bond types: single, double, aromatic). A third feature vector, "global state", is initialized as a randomized vector that is used to encode learned features of the whole polymer, and a connectivity matrix is generated so neighboring atoms and bonds can be referenced during message passing. (3) The atom, bond, global, and connectivity arrays are passed into the message passing blocks where a "learned" representation (*i.e.*, latent space embedding) of each atom and bond in the polymer is generated, which is a function of the local chemical environment of the atom or bond. Gilmer et al. provides more details in the implementation of graph neural networks for chemistry applications.[19] (4) The bond states are then passed through a dense layer to create a single bond vector where each value represents a bond in the polymer. (5) A global average pooling function is applied that averages this bond vector into a predicted value for the polymer property. By applying the global average pooling function, each bonds' value has a relative contribution to the predicted property. Therefore, a quantitative structure-property relationship can be inferred.

Figure S10 – Training loss. Exemplary loss and validation loss curves for training message passing neural network. Grey lines indicate individual models for the 10-fold cross-validation and red lines indicate average value across all 10 models.

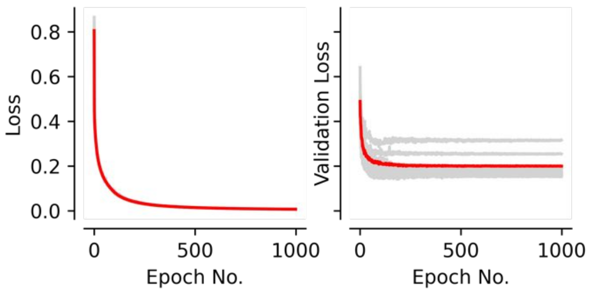

Present Addresses

[5] Department of Chemical Engineering, Stanford University, Stanford, CA 94305, USA
[6] Johnson & Johnson Vision, 7500 Centurion Parkway North, Jacksonville, Florida 32256, USA
[7] ExxonMobil Technology and Engineering Company, 1545 US-22 #1, Annandale, NJ 08801, USA
[8] Nvidia, 1881 9th Street #335, Boulder, CO 80302, USA

Supporting Information References

(1) Gurnani, R.; Kuenneth, C.; Toland, A.; Ramprasad, R. Polymer Informatics at Scale with Multitask Graph Neural Networks. *Chem. Mater.* **2023**, *35* (4), 1560–1567. https://doi.org/10.1021/acs.chemmater.2c02991.

(2) Kuenneth, C.; Rajan, A. C.; Tran, H.; Chen, L.; Kim, C.; Ramprasad, R. Polymer Informatics with Multi-Task Learning. *Patterns* **2021**, *2* (4), 100238. https://doi.org/10.1016/j.patter.2021.100238.

(3) Hevus, I.; Webster, D. C.; McNamara, J.; Ricapito, N. G.; Tymoshenko, S. Parallel Esterification of Bio-Based Dicarboxylic Acids in Small Scale Film Reactors: A High-Throughput Study. *Journal of Polymer Science* **2021**, *59* (8), 665–674. https://doi.org/10.1002/pol.20210059.

(4) Lim, B.-H.; Kwon, S.-H.; Kang, E.-C.; Park, H.; Lee, H.-W.; Kim, W.-G. Isolation and Identification of Cyclic Oligomers of the Poly(Ethylene Terephthalate)–Poly(Ethylene Isophthalate) Copolymer. *Journal of Polymer Science Part A: Polymer Chemistry* **2003**, *41* (7), 881–889. https://doi.org/10.1002/pola.10637.

(5) Gurnani, R.; Kuenneth, C.; Toland, A.; Ramprasad, R. Polymer Informatics At-Scale with Multitask Graph Neural Networks. September 27, 2022. https://doi.org/10.48550/arXiv.2209.13557.

(6) Kim, C.; Chandrasekaran, A.; Huan, T. D.; Das, D.; Ramprasad, R. Polymer Genome: A Data-Powered Polymer Informatics Platform for Property Predictions. *J. Phys. Chem. C* **2018**, *122* (31), 17575–17585. https://doi.org/10.1021/acs.jpcc.8b02913.

(7) Ma, R.; Liu, Z.; Zhang, Q.; Liu, Z.; Luo, T. Evaluating Polymer Representations via Quantifying Structure–Property Relationships. *J. Chem. Inf. Model.* **2019**, *59* (7), 3110–3119. https://doi.org/10.1021/acs.jcim.9b00358.

(8) Ramprasad, M.; Kim, C. Assessing and Improving Machine Learning Model Predictions of Polymer Glass Transition Temperatures. August 6, 2019. https://doi.org/10.48550/arXiv.1908.02398.

(9) Wu, S.; Kondo, Y.; Kakimoto, M.; Yang, B.; Yamada, H.; Kuwajima, I.; Lambard, G.; Hongo, K.; Xu, Y.; Shiomi, J.; Schick, C.; Morikawa, J.; Yoshida, R. Machine-Learning-Assisted Discovery of Polymers with High Thermal Conductivity Using a Molecular Design Algorithm. *npj Comput Mater* **2019**, *5* (1), 1–11. https://doi.org/10.1038/s41524-019-0203-2.

(10) Doan Tran, H.; Kim, C.; Chen, L.; Chandrasekaran, A.; Batra, R.; Venkatram, S.; Kamal, D.; Lightstone, J. P.; Gurnani, R.; Shetty, P.; Ramprasad, M.; Laws, J.; Shelton, M.; Ramprasad, R. Machine-Learning Predictions of Polymer Properties with Polymer Genome. *Journal of Applied Physics* **2020**, *128* (17), 171104. https://doi.org/10.1063/5.0023759.

(11) Zhang, Y.; Xu, X. Machine Learning Glass Transition Temperature of Polymers. *Heliyon* **2020**, *6* (10), e05055. https://doi.org/10.1016/j.heliyon.2020.e05055.

(12) Kim, C.; Batra, R.; Chen, L.; Tran, H.; Ramprasad, R. Polymer Design Using Genetic Algorithm and Machine Learning. *Computational Materials Science* **2021**, *186*, 110067. https://doi.org/10.1016/j.commatsci.2020.110067.

(13) Kuenneth, C.; Schertzer, W.; Ramprasad, R. Copolymer Informatics with Multitask Deep Neural Networks. *Macromolecules* **2021**, *54* (13), 5957–5961. https://doi.org/10.1021/acs.macromol.1c00728.

(14) Tao, L.; Varshney, V.; Li, Y. Benchmarking Machine Learning Models for Polymer Informatics: An Example of Glass Transition Temperature. *J. Chem. Inf. Model.* **2021**, *61* (11), 5395–5413. https://doi.org/923.

(15) Kuenneth, C.; Lalonde, J.; Marrone, B. L.; Iverson, C. N.; Ramprasad, R.; Pilania, G. Bioplastic Design Using Multitask Deep Neural Networks. *Commun Mater* **2022**, *3* (1), 1–10. https://doi.org/10.1038/s43246-022-00319-2.

(16) Moriwaki, H.; Tian, Y.-S.; Kawashita, N.; Takagi, T. Mordred: A Molecular Descriptor Calculator. *Journal of Cheminformatics* **2018**, *10* (1), 4. https://doi.org/10.1186/s13321-018-0258-y.

(17) Mordred, 2023. https://github.com/mordred-descriptor/mordred (accessed 2023-03-30).

(18) Pedregosa, F.; Varoquaux, G.; Gramfort, A.; Michel, V.; Thirion, B.; Grisel, O.; Blondel, M.; Prettenhofer, P.; Weiss, R.; Dubourg, V.; Vanderplas, J.; Passos, A.; Cournapeau, D.; Brucher, M.; Perrot, M.; Duchesnay, É. Scikit-Learn: Machine Learning in Python. *Journal of Machine Learning Research* **2011**, *12*.

(19) Gilmer, J.; Schoenholz, S. S.; Riley, P. F.; Vinyals, O.; Dahl, G. E. Neural Message Passing for Quantum Chemistry. **2017**. https://doi.org/10.48550/arXiv.1704.01212.
